# Designing and Evaluating Bayesian Advanced Adaptive Randomised Clinical Trials: A Practical Guide

**DOI:** 10.1002/pst.70042

**Published:** 2025-10-09

**Authors:** Anders Granholm, Aksel Karl Georg Jensen, Theis Lange, Anders Perner, Morten Hylander Møller, Benjamin Skov Kaas‐Hansen

**Affiliations:** ^1^ Department of Intensive Care 4131 Copenhagen University Hospital – Rigshospitalet Copenhagen Denmark; ^2^ Section of Biostatistics, Department of Public Health University of Copenhagen Copenhagen Denmark; ^3^ Department of Clinical Medicine, Faculty of Health and Medical Sciences University of Copenhagen Copenhagen Denmark

**Keywords:** adaptive trials, Bayesian statistics, randomisation, randomised clinical trial, response‐adaptive randomisation, simulation, trial design

## Abstract

Advanced adaptive randomised clinical trials are increasingly used. Compared to their conventional counterparts, their flexibility may make them more efficient, increase the probability of obtaining conclusive results without larger samples than necessary, and increase the probability that individual participants are allocated to more promising interventions. However, limited guidance is available on designing and evaluating the performance of advanced adaptive trials. Here, we summarise the methodological considerations and provide practical guidance on the entire workflow of planning and evaluating advanced adaptive trials using adaptive stopping, adaptive arm dropping, and response‐adaptive randomisation within a Bayesian statistical framework. This comprehensive practical guide covers the key methodological decisions for Bayesian advanced adaptive trials and their specification and evaluation using statistical simulation. These considerations include interventions and common control use; outcome type and generation; analysis timing and outcome‐data lag; allocation rules; analysis model; adaptation rules for stopping and arm dropping; clinical scenarios assessed; performance metrics; calibration; sensitivity analyses; and reporting. The considerations are covered in the context of realistic examples, along with simulation code using the *adaptr* R package. In conclusion, this practical guide will help clinical trialists, methodologists, and biostatisticians design and evaluate Bayesian advanced adaptive trials.

## 1 Introduction

Randomised clinical trials (RCTs) constitute the most rigorous research design for unbiased comparative effectiveness estimates of healthcare interventions [1]. However, conventional RCTs are limited by their inflexibility [1, 2]. Most conventional RCTs use a fixed maximum sample size with no or few interim analyses. Sample size calculations often rely on over‐optimistic assumptions [3, 4, 5, 6], which poses the risk that trials will be unable to provide firm conclusions about smaller, yet clinically relevant effects [1, 2]. Unfortunately, such results may incorrectly be interpreted as no difference between the interventions [7, 8, 9]. Also, RCTs may run longer than necessary, which may delay implementation of superior interventions or delay de‐implementation of inferior interventions, and thus result in slower improvements in patient outcomes and wasted research funding [1, 2, 10, 11]. Finally, except when stopped early, results are typically not used until the RCT has enrolled the planned maximum sample size, precluding continuous learning during the course of the trial [10, 12].

Adaptive trials use results from adaptive (interim) analyses to modify some aspects of the trial before completion, *without* undermining the integrity and validity of the trial [13]. The most common adaptive trials are *group sequential designs* [13], but they typically use only a few interim analyses with strict thresholds for early stopping [2]. There is an increased use of advanced adaptive trials (including adaptive platform trials) [10, 14, 15], that is, adaptive designs that often use multiple adaptive features and often involve many more adaptive analyses than conventional group sequential trials [1, 2, 13].

Advanced adaptive trials may be stopped entirely, or specific arms dropped, for several reasons—for example, inferiority/superiority, practical equivalence, futility, or at a pre‐specified maximum sample size—shortening the time required to reach valid and conclusive results [2, 10]. Adaptive arm dropping prioritises allocation to more promising interventions and increases power for the remaining comparisons when inferior arms are dropped early in trials with > 2 arms [2, 10]. Response‐adaptive randomisation increases allocation to arms more likely to be superior *before* the evidence is sufficient for overall termination of the trial [2, 10]. Both features can increase the probability of beneficial outcomes for randomised participants as the trial progresses [2, 10].

The increased flexibility, however, comes at a cost: everything else being equal, more adaptive analyses increase the risk of stopping or adapting due to chance findings. Further, while response‐adaptive randomisation may increase the chances of better outcomes for individual participants, it may also increase the overall required sample sizes in some cases and, unless adequately restricted, adaptations to random fluctuations may substantially impair trial performance either on average or in the worst‐case scenario [2, 16, 17, 18, 19, 20, 21, 22, 23]. As such, there have been ethical arguments both in favour of and against increased adaptation, especially response‐adaptive randomisation [20, 21, 22, 23, 24].

Consequently, it is paramount that the performance of advanced adaptive trial designs is carefully evaluated prior to initiation [1, 2, 13], and this is generally required by the competent authorities [25, 26]. In contrast to conventional non‐adaptive and less advanced adaptive trials such as two‐armed group sequential trials [13], this cannot be achieved with simple closed‐form sample size calculations and readily available methods for defining stopping rules. Instead, statistical simulation is required [2, 25, 26, 27, 28]. Planning advanced adaptive trials may hence seem daunting due to the more comprehensive processes that require specific methodological and statistical competences.

To alleviate this, we provide a practical guide covering the entire process of planning and evaluating Bayesian advanced adaptive trials using the *adaptr* [27] R package. We provide guidance on how to use simulations to evaluate and compare advanced adaptive designs and examples of the code required to do so.

## 2 Overview

### 2.1 Scope and Target Audience

The key phases in an RCT are (1) identification of the clinical problem and formulation of the research question; (2) trial design; (3) trial conduct; and (4) analysis and reporting. The scope of this manuscript is to provide an example‐based practical guide on key steps necessary for designing Bayesian advanced adaptive trials, including the use of simulations for assessing their performance (e.g., expected sample sizes, type 1 error rates, power, etc.), corresponding to those parts of the second phase that are specific to advanced adaptive trials. As such, methodological considerations relevant regardless of the adaptations (e.g., setting, number of centres, use of blinding, procedures for inclusion and follow‐up, etc.) are not covered here. We focus on phase 3 or 4 comparative effectiveness trials with adaptive stopping, arm dropping, and/or response‐adaptive randomisation using a Bayesian framework, as is common in advanced adaptive trials [10, 14]. We intentionally do not make any comparisons with other trial designs, for example, fixed or frequentist adaptive designs, to keep focus and limit the extent of this guide. However, comparisons with such designs may be relevant in practice, and these designs come with other advantages and disadvantages with regard to performance metrics as well as operational aspects and implementation.

The target audience is clinical trialists, methodologists, and biostatisticians with previous experience on RCT planning and conduct and, ideally, basic knowledge of Bayesian statistics, adaptive trial designs, and the R statistical software. Additional guidance and information on other aspects of adaptive trial planning and conduct can be found elsewhere [2, 10, 13], including the *PANDA* [panda.shef.ac.uk] and *CTTI* [ctti‐clinicaltrials.org/our‐work/novel‐clinical‐trial‐designs] repositories.

### 2.2 Bayesian Statistics and Advanced Adaptive Trials

**FIGURE 1:**
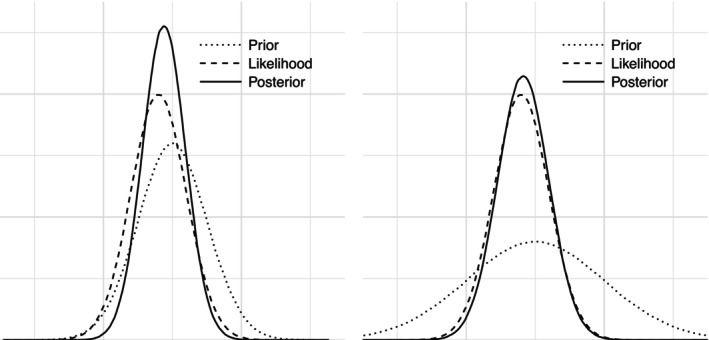
Illustration of probabilities in Bayesian analyses. Horizontal axes represent specific values on the outcome scale (on a fictive, unitless scale in this example), while vertical axes represent densities (with higher values being more probable). Posterior probability distributions (full lines) combine prior probability distributions (dotted lines) with the obtained data through a likelihood function (dashed lines), with the posterior probability being a weighted compromise between the prior and the data. The left subplot illustrates a relatively informative (narrow) prior distribution with the resulting posterior centred between the prior and the likelihood, but more precise (i.e., narrower) than both. The right subplot illustrates a less informative (wider) prior, where the posterior largely overlaps with the likelihood and is only slightly more precise than the likelihood itself. The data and likelihood functions are identical in both subplots.

Within a Bayesian statistical framework, uncertainty is expressed using probability distributions [29]. In brief, results are expressed as a *posterior* probability distribution that is a weighted compromise between a *prior* probability distribution reflecting the belief before obtaining the new data and the observed data expressed via a likelihood function [29], as illustrated in Figure 1.

In this context, the term *advanced adaptive trials* refers to adaptive trials with more adaptations or more types of adaptations than conventional group sequential trials. Advanced adaptive trial designs are not restricted to using a Bayesian approach, despite this being the focus of this text. However, the Bayesian statistical approach is well suited for advanced adaptive trial designs as the implementation and evaluation of the adaptation rules are relatively simple once the posterior distributions are available. Of note, many Bayesian adaptive trials may technically be considered hybrid Bayesian‐frequentist, as Bayesian analogues of inherently frequentist concepts such as type 1 error rates and power are evaluated using long‐run frequencies from statistical simulations [2, 30]. While the importance of *always* tightly controlling these metrics has been discussed [31, 32], the competent authorities typically require this for late‐phase trials [25, 26], and so may funders and ethical committees [33]. It is thus usually recommended and done [16, 34], as it is otherwise difficult to ensure that the adaptive features do not challenge the validity of the trial [13].

### 2.3 Contents and Software

The rest of the guide summarises the relevant key methodological considerations and covers how trial designs are specified and evaluated with simulations using the *adaptr* [27] R package, including sensitivity analyses, and covers the reporting of results. Finally, a brief discussion is provided covering limitations with the described approach and the package.

The *adaptr* [27] package simulates adaptive (multi‐arm, multi‐stage) RCTs using adaptive stopping and arm dropping for superiority, inferiority, practical equivalence, and/or futility, as well as fixed and/or response‐adaptive randomisation. We used *adaptr* v1.4.0 with R v4.4.1 for this guide; complete details on simulation options, arguments, and additional functions in *adaptr*, including visualisation functions, can be found in the package documentation (inceptdk.github.io/adaptr). Of note, a number of different software solutions for the simulation of adaptive multi‐arm, multi‐stage RCTs exist; these come with different functionality, advantages, and disadvantages, as summarised elsewhere [28, 33]. Different software solutions may be preferable depending on the research question and intended design. Herein, we use the *adaptr* package for several reasons. First, it is open‐source, freely available, flexible, extensive, well‐documented, and relatively fast. Second, the use of R code (as opposed to simulation software with graphical user interfaces) facilitates reproducibility and makes it easy to follow every step of the tutorial.

### 2.4 Regulatory Requirements

As for all RCTs, adherence to regulatory requirements and guidance from the relevant competent authorities (e.g., the European Medicines Agency [25] or the United States Food and Drug Administration [26, 35]) is necessary. This guide does not specifically focus on regulatory requirements, as these will vary according to the purpose of the trial (e.g., whether the purpose is to obtain marketing authorisations for new drugs or to provide comparative effectiveness data for interventions already approved and in use) and between jurisdictions. Approval of advanced adaptive trials using design features covered in this guide will generally require clear pre‐specification of adaptation rules, thorough evaluation of performance metrics, and acceptable type 1 error rates; these issues are covered in the relevant sections of this guide.

## 3 Methodological Choices and Simulation‐Based Evaluation

**FIGURE 2:**
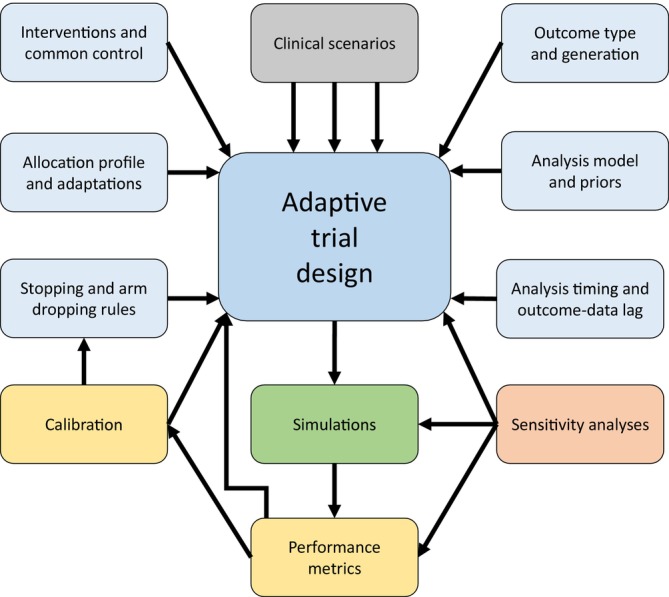
Overview of the process of designing and evaluating Bayesian advanced adaptive trials, with focus on methodological considerations related to the trial design, the simulation process, measurement of performance metrics (e.g., expected sample sizes, type 1 error rates, power, etc.), calibration, iterative revisions of the trial design, and finally, the use of sensitivity analyses to assess the influence of different design choices and assumptions on performance. Light blue boxes cover essential design choices that may be varied, but where the final design will only be based on a single choice for each option; *clinical scenarios* (grey box) are similarly essential, but generally, multiple scenarios will be evaluated for each single combination of all other design choices. Simulations (green box) are used to evaluate trial designs (i.e., calculate performance metrics) across different clinical scenarios and to optionally *calibrate* stopping rules to obtain acceptable values for one or more performance metrics (both in yellow boxes). Finally, sensitivity analyses of design choices and uncontrollable assumptions (orange box) will typically be used to evaluate the implications of different design choices and other plausible assumptions on performance metrics through simulations. The figure is inspired by a figure in a previous article by our group [2]; additional guidance on the methodological choices may also be found in that article.

The key methodological considerations that we cover are presented in Figure 2. Importantly, methodological decisions interact, and the development and evaluation of an advanced adaptive trial design will typically be an iterative process [2].

The following sections describe the key methodological choices in general, along with details on their specific implementation in *adaptr* and an example including code, mostly in that order. The example trial uses three interventional arms without a common control arm, an undesirable binary outcome, restricted response‐adaptive randomisation, stopping and arm dropping for superiority/inferiority and practical equivalence, and a maximum sample size of 10,000 participants. While the example is not based on any specific trial, it could realistically correspond to a large, pragmatic RCT conducted in adult intensive care unit patients with new‐onset atrial fibrillation, assessing three already approved and commonly used drugs for this condition (amiodarone, beta blockers, and digoxin) [36, 37] with all‐cause mortality as the primary and *guiding* outcome. Complete code and all outputs from the primary example are included in Appendices A and B in Supporting Information.

### 3.1 Setup

First, load *adaptr*, set up a cluster for parallel computation for faster simulations, and define where results are saved:**library**(adaptr)**setup_cluster**(10) *# Number of cores for parallel computation*dir_out <- "<PATH>/" *# Replace with an actual, permanent path*


### 3.2 Trial Design

In *adaptr*, trial designs including outcome generation and analysis models are specified via the *setup_trial()* function in the general case or one of its special‐case variants. For binary, binomially distributed outcomes, *setup_trial_binom()* can be used and has very weak, flat priors (*Beta(alpha = 1, beta = 1)* priors, which correspond to two randomised participants, one with the outcome and one without [38]). For continuous, normally distributed outcomes, *setup_trial_norm()* can be used and uses no prior information. Our primary example uses *setup_trial_binom()*. In the following, we only include the arguments specifying the options discussed in each section (with omitted parts of the code marked with *…*). Code for the complete trial design is specified at the end of this section. We explicitly specify certain key arguments for clarity, even when identical to the defaults.

#### 3.2.1 Interventions and Use of Common Control

The initial trial interventions (arms) must be specified, and a *common control* arm may be specified if relevant; the use of a common control arm will influence trial behaviour for multi‐arm designs. Without a common control arm, all adaptive decisions will be based on the probabilities of each arm being overall best or of all arms being practically equivalent. With a common control arm, all other arms will be compared *pairwise* against it, with all stopping/arm‐dropping decisions based on probabilities from the pairwise comparisons (Section 3.2.6). If a non‐control arm is superior, the current common control arm is dropped, and the superior arm is promoted to the new control arm. This is followed by immediate pairwise comparisons against the remaining non‐control arms before the inclusion of additional simulated participants. If multiple non‐control arms are superior, the one with the highest probability of being overall best is chosen. Even when one arm represents the *standard of care*, we advise that designs both with and without a formal common control arm are assessed due to the influence of this decision on a trial design's performance.

Here, we include three arms without a formal common control, as none of the three interventions in the example can be considered to represent the standard of care. To keep the code generic (and, for the purpose of the simulations conducted in this example, it does not matter which arm is which, as there is no common control arm), the arm names are generic:**setup_trial_binom**( arms = **c**("Arm A", "Arm B", "Arm C"), control = NULL, …)


#### 3.2.2 Outcome Type and Generation

A single *guiding* outcome is simulated and used for all adaptations. Typically, this will be the primary trial outcome, but an *intermediary* or *surrogate* outcome, for example, the same outcome after a shorter follow‐up period or another outcome that is highly correlated with the primary outcome, may be chosen based on careful considerations [2, 13, 39]. To generate outcome data, the outcome distribution must be defined. Advanced adaptive trial designs should be assessed under multiple different clinical *scenarios*, that is, different sets of assumed outcome distributions in each arm, which must be specified as part of separate but otherwise identical trial specifications (further details in Section 3.2.7). We recommend initially specifying a scenario without differences present and using the clinically most plausible outcome distribution as the reference.

In *adaptr*, outcomes must be numerical, *even* if they correspond to, for example, binary or ordinal outcomes. Further, we must specify whether higher or lower values are desirable. The example uses an undesirable, binary, binomially distributed outcome—mortality—encoded to reflect the common encoding of mortality: 0 denotes survival (no event) while 1 denotes death (event). Here, the (assumed) true event probabilities are 25% in all arms, reflecting no between‐arm differences:**setup_trial_binom**( … true_ys = **c**(0.25, 0.25, 0.25), highest_is_best = FALSE, …)


#### 3.2.3 Analysis Timing and Outcome‐Data Lag

The number of participants analysed at the time of each adaptive analysis must be specified along with an adequately large *burn‐in*: an initial period where no adaptations occur before a sufficient number of participants are included. This prevents adaptations to early, random fluctuations [2, 18, 24, 39] and avoids stopping trials or dropping arms with samples so small that results may be considered unreliable or that the precision of effects on important outcomes (e.g., safety outcomes) may be considered too low. The choice of the size of the burn‐in period should thus consider the precision for the guiding/primary outcome and important additional outcomes. The consequences of different burn‐in periods can be assessed in sensitivity analyses of design choices (Section 3.7), as the effects may not be obvious: for example, a longer burn‐in will increase the minimum sample size, but may limit the maximum number of analyses and the risk of adaptations to random fluctuations early, and thus the effects on the expected sample sizes are difficult to determine without simulation.

Importantly, in trials with small maximum sample sizes, the benefit of adaptations may not outweigh the advantages, as the precision of estimates from adaptive analyses before the maximum may be limited, and the risk of random fluctuations driving adaptations may be too high. In these cases, fixed designs (or at least adaptive designs with few possibilities for adaptations, with restrictions to possible adaptations early) or the use of external information through prior probability distributions can be considered.

The timing of subsequent adaptive analyses should consider both the maximum total number of analyses, the maximum allowed sample size, and the expected inclusion rates. While more analyses mean that stricter stopping thresholds may be required (Section 3.2.6), a lower number of analyses will limit the potential benefits of using an adaptive design [2, 24].

Importantly, the outcome‐data lag and the expected inclusion rate should be considered, as both will affect the efficiency and reliability of adaptations and ultimately the performance metrics [40]. Outcome‐data lag is the outcome follow‐up duration plus the expected time required to obtain, clean, and validate data before analyses can be conducted [40]. The expected inclusion rate can be constant over time or change, for example, if the number of active trial sites is expected to change. Longer outcome‐data lags or higher inclusion rates mean that the proportion of randomised participants with data available at the time of each adaptive analysis will be lower, increasing the risk that results will change (in direction or magnitude) at the final analysis conducted after stopping enrolment and completing follow‐up for all randomised participants [40, 41]. It has been suggested that the ratio between the outcome follow‐up duration and the expected inclusion period should be < 0.25 for adaptive trials to be beneficial [42]. In previous trials by our group, inclusion rates were mostly constant after initiation of all participating trial sites [43, 44, 45, 46, 47], but this will vary between trials.

Ideally, the maximum sample size should ensure that there is an acceptable probability of conclusiveness (i.e., ultimately triggering one of the decision rules for superiority/inferiority, practical equivalence, or futility) to ensure that the trial will provide useful results. As the maximum sample size will also be influenced by practical and economic considerations, other design choices may have to be varied until an acceptable compromise can be made. Some advanced adaptive trials [48] are planned *without* a maximum sample size; these can be simulated using *adaptr* by setting an implausibly high maximum sample size and ensuring that a stopping rule will always be triggered before the specified sample size limit (Section 3.2.6). Analysis timing is typically based on the number of participants that have completed their outcome‐data lag period and can be included in the analysis and not on the total number of participants randomised.

Below, we specify that the first analysis will be conducted after 500 participants have available data, with subsequent analyses after each 250 additional participants up to a maximum sample size of 10,000 participants. The example assumes a constant lag of 200 participants due to a constant assumed inclusion rate:**setup_trial_binom**( …*# Number of participants with data available and included in each analysis* data_looks = **seq**(from = 500, to = 10000, by = 250),*# Number of participants randomised at each analysis* randomised_at_looks = **c**(**seq**(from = 700, to = 9950, by = 250), 10000),*# Note: the maximum number in both arguments should be equal* …)


#### 3.2.4 Allocation Profiles

The initial allocation profile and subsequent use of fixed allocation, response‐adaptive randomisation, or combinations must be specified, including any restrictions. Although response‐adaptive randomisation increases the probability of allocating more participants to more promising interventions [2, 16, 23], there are ethical, practical, and logistical arguments both in favour and against its use [20, 21, 22, 23, 24, 49, 50].

Response‐adaptive randomisation affects trial performance (Section 3.3) differently depending on the number of arms, whether a common control arm is used, and whether between‐arm differences are present [2, 17, 18, 19, 20, 21, 23, 40, 51, 52]. Previous results indicate that fixed allocation or relatively restricted response‐adaptive randomisation may be preferable in two‐arm trials; both fixed and relatively restricted response‐adaptive allocation may perform well in trials with > 2 arms with no common control; and a relatively higher, fixed allocation probability to the control arm and response‐adaptive randomisation to non‐control arms may be preferable in trials with > 2 arms and a common control [2, 33]. Importantly, response‐adaptive randomisation may improve certain performance metrics while worsening others: higher probabilities of desirable outcomes for individual participants, for example, may on average require larger samples [2, 17, 18, 19, 20, 21, 23, 40, 51, 52]. Further, even when response‐adaptive randomisation on average improves performance, it may cause poorer performance in the *worst‐case* scenario due to adaptations to random fluctuations that can take time to reverse [2]. Potential negative implications may be mitigated by restricting the response‐adaptive randomisation [2, 33]. Response‐adaptive randomisation may be restricted in two ways. First, by imposing minimum and maximum allocation probabilities, which may be rescaled when arms are dropped. Second, by *softening*, that is, raising the raw allocations probabilities to some exponent (the softening factor), which could be between 0 (leading to equal allocation probabilities after rescaling) and 1 (no restriction), most commonly between 0.5 and 1.0 [2, 53]. With a common control arm, a relatively higher and possibly fixed allocation probability to the control arm may increase statistical power [2, 39].

When response‐adaptive randomisation is used, operational complexity is increased by the need to handle potential *time drift*. Time drift is the potential bias due to changes between periods in the included population or concurrent interventions used with different allocation probabilities [2, 10, 23, 33, 54]. Similarly, stratified block randomisation to balance important prognostic factors is difficult to combine with response‐adaptive randomisation [2, 55]. For these reasons, using an adequate *burn‐in* period (possibly using stratified block randomisation) before allowing response‐adaptive randomisation *and* restricting the response‐adaptive randomisation is advisable.

In *adaptr*, response‐adaptive randomisation is based on each arm's overall probability of being best [2, 23, 56]. Softening factors can vary across adaptive analyses, to ensure, for example, equal allocation or more restrictive response adaptivity early in the trial. *adaptr* supports multiple specific control‐arm allocation rules: ratios of 1 (for each non‐control arm) to the square root of the number of non‐control arms (for the control arm) [2, 39, 57] or a control‐arm allocation probability equal to the highest probability among the non‐control arms [2, 18]. Fixed allocation probabilities may be used for some or all arms. Here, the example trial will initially use equal allocation probabilities of 33.3% to each arm, followed by response‐adaptive randomisation with restrictions in the form of 25% minimum limits that will be rescaled when an arm is dropped, and a softening factor of 0.5:**setup_trial_binom**( … start_probs = **c**(1**/**3, 1**/**3, 1**/**3), fixed_probs = NULL, min_probs = **c**(0.25, 0.25, 0.25), rescale_probs = "limits", soften_power = 0.5, …)


#### 3.2.5 Analysis Model and Priors

The statistical model, including priors, for the primary outcome in the *actual* trial should guide the selection of the statistical model used in simulations, although it is common and acceptable to simplify both the model and the estimation method. For example, adjusting for important covariates during simulations is complex and usually omitted, akin to conventional sample size calculations [2], and simulations may use conjugate models [27, 58] instead of full Markov chain Monte Carlo estimation to ensure speed and feasibility.

Importantly, the prior distributions should be adequately justified, particularly if informative priors incorporating previous, external evidence are used [35], as the priors will influence both the performance metrics of the trial design and the interpretation of the final trial. Non‐ or weakly informative priors may be used if there is either substantial uncertainty, or if no influence of previous data on the results is wanted. Alternatively, neutral (i.e., not favouring any intervention), informative (‘sceptical’) priors may be used to limit the risk of erroneous conclusions [51].

*adaptr* supports different models and modelling approaches and only requires that draws from the posterior probability distributions are returned for each trial arm on the natural (absolute) scale for the outcome of interest [27]. Thus, for binary outcomes, posterior draws should reflect event probabilities in each arm. The number of posterior draws used in each arm should be adequate to compare trial arms; if, for example, stopping thresholds are calibrated (Section 3.5), a larger number may be required, as it determines the granularity of the estimated probabilities (e.g., with 1000 posterior draws, the minimum non‐zero difference in probabilities is 0.1%‐points). Thus, we use 10,000 posterior draws in the example. As we use *setup_trial_binom()*, we do not manually have to specify an analysis model or priors; conjugate beta‐binomial models with flat priors are used: [38, 58]**setup_trial_binom**( … n_draws = 10000, …)


#### 3.2.6 Stopping and Arm Dropping Rules

**FIGURE 3:**
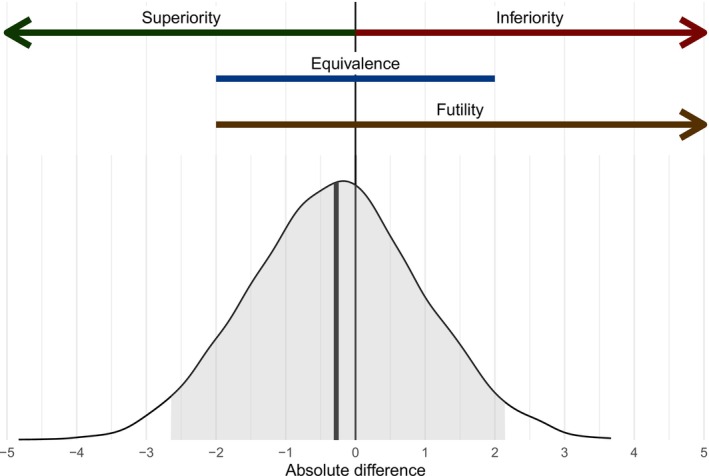
Illustration of probabilistic decision rules for a single two‐arm comparison with an undesirable outcome (i.e., negative differences are preferable). The lower part of the figure shows the posterior probability distribution on the absolute scale (e.g., %‐points in the example used in the text) with the median value highlighted by the vertical bold line and the 95% percentile‐based credible interval highlighted in grey. The upper part of the figure illustrates how the posterior is partitioned to calculate the probabilities of superiority, inferiority, practical equivalence, and futility, which are simply the proportion of posterior samples in each ‘*region’* of interest. Figure based on a similar figure previously presented elsewhere [2].

A maximum sample size must be specified for simulations and will affect multiple performance metrics, including type 1 error rates and power [2]. Stopping and arm‐dropping rules for superiority, inferiority, practical equivalence, and futility may be specified in *adaptr* [2, 27], as illustrated in Figure 3.

Stopping rules for superiority/inferiority are mandatory and have the highest priority, that is, they will be assessed *before* stopping rules for practical equivalence or futility, as concluding that an arm is superior is more clinically useful than, for example, a futility decision. In *adaptr*, without a common control, stopping/arm‐dropping rules for both superiority and inferiority are based on each remaining arm's probability of being overall superior (i.e., the best amongst all remaining arms). With a common control, probability thresholds relate to pairwise comparisons with the common control arm, with inferior non‐control arms dropped and superior control arms leading to the common control being dropped, with the superior non‐control arm taking the role of the common control in future comparisons [2, 10].

Although stopping rules may be set so as to be sufficient to change clinical practice [2, 59], regulatory bodies will typically require type 1 error rates ≤ 5% [2, 25, 26, 35]. This can be achieved by manual iteration or automatic calibration (Section 3.5). Stopping thresholds for superiority and inferiority are usually symmetric (i.e., the decision threshold for inferiority is defined as 100% minus the decision threshold for superiority) and may either be *constant* throughout the trial or more conservative at earlier analyses. The former correspond to Pocock monitoring boundaries and the latter to, for example, O'Brien Fleming monitoring boundaries in conventional group sequential trial designs [33, 60]. Constant decision thresholds generally lead to smaller expected sample sizes, lower errors in estimates, and less overestimation of intervention effects when stopped early, but lower power compared to varying, decreasingly strict decision thresholds [61, 62, 63]. Consequently, the latter are often favoured in conventional trials *expected* to run until the maximum sample size and mainly use interim analyses as a safety measure, while constant decision thresholds may be preferable in advanced adaptive trials *not* expected to run until the maximum sample size.

Optional stopping rules for practical equivalence may be defined and will be evaluated *after* superiority/inferiority [2, 27]. Without a common control, the entire trial will be stopped if the largest absolute difference between all active arms is smaller than a pre‐specified threshold with a sufficiently high probability, for example, > 90% probability that the largest absolute difference is < 2.5%‐points [2, 27]. With a common control arm, non‐control arms will be dropped for equivalence if the absolute difference compared to the common control is smaller than a pre‐specified threshold with sufficiently high probability; the overall trial will be stopped if only the common control arm remains.

Optional stopping rules for futility may be defined when a common control arm is used and will be evaluated *after* all other stopping rules. Of note, futility may also be assessed against external, for example, historical, controls, but assessment against historical controls only is not supported in *adaptr* (although historical data may be combined with concurrent controls through the priors used) and comes with challenges discussed elsewhere [64]. Non‐control arms will then be dropped for futility if the probability that they are *not* sufficiently *better* than the control is above a pre‐specified threshold, for example, > 90% probability of a *beneficial* difference is < 2.5%‐points, including the probability of the non‐control arm being worse [2, 27]. The overall trial will be stopped if only the control remains.

In *adaptr*, all probability thresholds may vary across analyses and can be stricter in earlier analyses and more lenient in later analyses. By setting thresholds to either 100% or 0% (which will never be exceeded), stopping rules can be disabled at early analyses, making it possible to only use *some* stopping rules early or to use response‐adaptive randomisation before allowing stopping or arm dropping. Of note, the *differences* of interest on the absolute scale for practical equivalence and futility stopping rules must be constant. Probability thresholds for practical equivalence and futility may be manually or automatically calibrated to obtain specific performance metrics, but this is optional. Probability thresholds for practical equivalence and futility may be lower than the corresponding superiority/inferiority thresholds, as they will otherwise often require substantially more participants to be triggered [2]. Of note, allowing trials to stop for practical equivalence or futility may decrease the probability of stopping for superiority and may thus reduce power while increasing the probability of a conclusive result, that is, triggering any stopping rule [2]. With a common control, practical equivalence and/or futility may be evaluated only against the *first* control arm (as will often be most relevant) or also against any other arms that are subsequently promoted to controls.

Finally, if no maximum sample size is desired for the actual trial and an artificially high maximum sample size is specified (Section 3.2.3), the chosen stopping rules must lead to probabilities of triggering a stopping rule of 100% across all evaluated scenarios and sensitivity analyses of assumed parameters (Section 3.8) to ensure valid estimates of trial design performance.

Here, we specify constant inferiority and superiority stopping thresholds and a stopping rule for equivalence that first becomes active when data from 1500 participants are analysed. Following this, the trial stops if there is > 90% probability that the largest absolute difference in mortality between all remaining arms is < 2.5%‐points:**setup_trial_binom**( … inferiority = 0.01, superiority = 0.99, equivalence_prob = **ifelse**(**seq**(from = 500, to = 10000, by = 250) **<** 1500, 1, 0.9), equivalence_diff = 0.025, …)


#### 3.2.7 Clinical Scenarios

Typically, advanced adaptive trial designs are evaluated under multiple clinical scenarios, for example, assuming different arm‐specific outcome distributions and thus different intervention effects [2]. Trial designs should be evaluated using a so‐called *null* scenario *without* between‐arm differences and at least one scenario *with* between‐arm differences (of note, for designs with > 2 arms, these may include scenarios where all arms are different or where only one or more arms are different from the others) [2]. The probability of ultimately stopping for superiority in the *null* scenario corresponds to the type 1 error rate in this scenario [2, 18, 24, 26, 65]; although type 1 error in other scenarios may also be assessed for trial designs with > 2 arms, as described in Section 3.3. Automatic calibration will typically use the *null* scenario (Section 3.3). The primary *null* scenario should be based on the most likely *reference* outcome distribution, for example, the most likely event probability based on existing clinical knowledge.

The probabilities of stopping for superiority in scenarios with differences are used to assess *power* [2] and other performance metrics, and may also be used to assess type 1 error rates in trial designs with > 2 arms (Section 3.3). We recommend using multiple scenarios with combinations of no difference and at least two different magnitudes of differences present, for example, *small* and *large* differences, with at least one arm using the same outcome distribution as the primary *null* scenario. Small differences might be aligned with the thresholds used for equivalence or futility, and ideally correspond to the minimally relevant difference. Large differences might correspond to the anticipated or largest expected realistic intervention effect [2].

Practically, each scenario is expressed in *adaptr* as one trial design specification with different outcomes in each arm and all other design choices being identical across scenarios, which are then evaluated. The full code to specify the primary *null scenario* is shown here and combines the code snippets presented so far with fewer comments:primary_design_null_scenario <- **setup_trial_binom**(*# Arms and scenario* arms = **c**("Arm A", "Arm B", "Arm C"), control = NULL, true_ys = **c**(0.25, 0.25, 0.25), highest_is_best = FALSE,*# Allocation rules* start_probs = **c**(1**/**3, 1**/**3, 1**/**3), fixed_probs = NULL, min_probs = **c**(0.25, 0.25, 0.25), rescale_probs = "limits" , soften_power = 0.5,*# Participants with data available/randomised at each analysis* data_looks = **seq**(from = 500, to = 10000, by = 250), randomised_at_looks = **c**(**seq**(from = 700, to = 9950, by = 250), 10000),*# Stopping rules* inferiority = 0.01, superiority = 0.99, equivalence_prob = **ifelse**(**seq**(from = 500, to = 10000, by = 250) **<** 1500, 1, 0.9), equivalence_diff = 0.025,*# Posterior draws* n_draws = 10000)


### 3.3 Performance Metrics

Performance metrics of interest must be chosen and prioritised before simulating and comparing design variants. Different metrics (Table 1) may be preferred according to the research question and specific trial [2, 18, 24], and optimising one performance metric will often worsen other metrics.

**TABLE 1:** Performance metrics.

Performance metric	Description
Sample size	Total sample size (across arms) in each simulation. Summarised across simulations using means (i.e., expected values), SDs, medians, IQRs, and ranges. Lower sample sizes are preferable for economical/logistical reasons and to allow results to be used faster for future patients. Note that a low mean sample size does not rule out a small probability of a very large sample size; one should therefore look at all the sample size metrics.
Summed outcome data	Total summed outcome data (across all arms) in each simulation, that is, total event counts for binary outcomes (e.g., mortality) or total sums of continuous outcomes (e.g., days alive and out of hospital). Summarised across simulations using means, SDs, medians, IQRs, and ranges. Depending on whether an undesirable or desirable outcome is used, lower or higher values, respectively, are preferable for *internal* patients (trial participants).
Ratio of summed outcome data to sample size	Ratio of total summed outcome data to sample size (across all arms; summed outcome data divided by sample size) in each simulation, i.e., total event probabilities for binary outcomes (e.g., mortality) or overall means for continuous outcomes (e.g., days alive and out of hospital). Summarised across simulations using means, SDs, medians, IQRs, and ranges. Depending on whether an undesirable or desirable outcome is used, lower or higher values, respectively, are preferable for *internal* patients (trial participants).
Probabilities of conclusiveness, superiority, equivalence, futility, and stopping after the maximum number of adaptive analyses without triggering any stopping rule	The proportions of simulated trials stopped due to different stopping rules, i.e., superiority, practical equivalence, and futility. The probability of conclusiveness is the combined probability of stopping for either superiority, practical equivalence, or futility, while the probability of stopping after the maximum number of adaptive analyses without triggering any stopping rule corresponds to the probability of inconclusiveness. The probability of superiority may be interpreted as the Bayesian analogue to the type 1 error rate for scenarios containing no between‐arm outcome differences, and as the Bayesian analogue of the power for scenarios containing between‐arm differences [2] (for trials with > 2 arms, type 1 error rates may also be assessed in other scenarios with some arms identical and others being different, as described in the text). Higher probabilities of conclusiveness are preferred (especially with regard to *external*/future patients) to increase the usefulness of trial results, and higher probabilities of superiority may be desired at the expense of lower probabilities of equivalence or futility, as superiority decisions may be more clinically useful if a difference exists.
Probabilities of selecting different arms or no arm	The proportions of simulated trials selecting different arms or no arm after stopping (according to the arm selection strategy used [2], as described in the text). For both *internal* patients (participants) and *external*/future patients, higher probabilities of selecting arm(s) that are truly better are preferable.
Probabilities of erroneous overall superiority decisions	The proportions of simulated trials ultimately stopped for superiority, but where the arm declared as overall superior does not correspond to a single superior arm (i.e., declaring an inferior arm or an arm with at least another arm being exactly equivalent as superior will both be considered erroneous). This can correspond to the probabilities of type 1 errors regarding the overall conclusions across a number of scenarios with different assumed outcome distributions in arms.
Root mean squared error/median absolute error of the effect estimate in the selected arm	Root mean squared error (RMSE)/median absolute error (MAE) of the intervention effect estimate (e.g., the estimated event probability) for the selected arm in each simulation (according to the arm selection strategy used [2], as described in the text) across simulations compared to the ‘true’ simulated value across trials. Lower RMSEs/MAEs are preferable as this corresponds to higher accuracy. *Calculation*: Let *k* = 1, …, *K* denote each simulation where an arm has been selected according to the procedure described in Section 3.3. Define the error term in the effect estimate in the selected arm in each simulation with a selected arm as: $\varepsilon_{k}≔{\hat{x}}_{\text{selected}\left(k\right)}-x_{\text{selected}\left(k\right)}$ With ${\hat{x}}_{\text{selected}\left(k\right)}$ denoting the central estimate (mean or median) of the posterior distribution in the selected arm in simulation *k*, and $x_{\text{selected}\left(k\right)}$ denoting the true value (e.g., mean or event probability) in the selected arm in simulated *k*. With these definitions, the RMSE and MAE of the effect estimate across selected arms across simulations can be defined as: $\text{RMSE}=\sqrt{\frac{\sum_{k=1}^{K}{\varepsilon_{k}}^{2}}{K}}$ $\mathrm{MAE}=q_{50}\left(\left|\varepsilon_{k}\right|\right)$ Here, *q*_*50*_ denotes the 50%‐quantile of the empirical distribution of error terms.
Root mean squared error/median absolute error of the intervention effect	Root mean squared error (RMSE)/median absolute error (MAE) of the estimate of the intervention effect (the difference between the estimate for a selected non‐control arm compared to the estimate from a control or otherwise specified reference arm) compared to the ‘true’ intervention effect (the difference between the ‘true’ simulated value for the selected non‐control arm compared to the ‘true’ simulated value in a control/reference arm) across simulations. Lower RMSEs/MAEs are preferable as this corresponds to higher accuracy. Calculation depends on the arm selection strategy used [2], as described in the text. *Calculation*: Let *k* = 1, …, *K* denote each simulation where an arm *other* than the reference (e.g., the control arm) has been selected according to the procedure described in Section 3.3. Define the error term in the intervention effect estimate in the selected arm in each simulation with a non‐reference arm selected as: $\varepsilon_{k}≔\left({\hat{x}}_{\text{selected}\left(k\right)}-{\hat{x}}_{\text{reference},k}\right)-\left(x_{\text{selected}\left(k\right)}-x_{\text{reference}}\right)$ With ${\hat{x}}_{\text{selected}\left(k\right)}$ denoting the central estimate (mean or median) of the posterior distribution in the selected arm in simulation *k*; and ${\hat{x}}_{\text{reference},k}$ denoting the central estimate (mean or median) of the posterior distribution in the reference arm in simulation *k*; $x_{\text{selected}\left(k\right)}$ denoting the true value (e.g., mean or event probability) in the selected arm in simulation *k*; and $x_{\text{reference}}$ denoting the true value in the reference arm. With these definitions, the RMSE and MAE of the intervention effect across selected arms across simulations with a non‐reference arm selected can be defined as: $\text{RMSE}=\sqrt{\frac{\sum_{k=1}^{K}{\varepsilon_{k}}^{2}}{K}}$ $\mathrm{MAE}=q_{50}\left(\left|\varepsilon_{k}\right|\right)$ Here, *q*_*50*_ denotes the 50%‐quantile of the empirical distribution of error terms.
Ideal design percentage	The ideal design percentage (IDP) [2, 18] combines arm selection probabilities and the consequences of selecting different arms into a single measure (e.g., high probabilities of selecting an arm that is only slightly inferior to the best arm is less problematic than high probabilities of selecting an arm that is substantially worse than the best arm). Especially relevant when comparing trial designs with > 2 arms; higher values (closer to 100%) are preferable as this corresponds to increased benefit for *external*/future patients. Calculation depends on the arm selection strategy used [2] as described in the text; IDPs are not calculated for scenarios with no between‐arm differences. Mostly relevant and interpretable when comparing multiple designs. *Calculation*: Let *k* = 1, …, *K* denote each simulation where an arm has been selected according to the procedure described in Section 3.3. Define the expected outcome measure *E(y)* (e.g., mean or event probability) in patients outside the trial following trial completion as: $E\left(y\right)=\frac{1}{K}\sum_{k=1}^{K}x_{\text{selected}\left(k\right)}$ With $x_{\text{selected}\left(k\right)}$ denoting the true value (e.g., mean or event probability) in the selected arm in simulation *k*. Further, let *x*_*min*_ denote the lowest true outcome value (regardless of whether lower or higher values are best) across trial arms, and let *x*_*max*_ denote the highest true outcome value across the trial arms. The IDP can then be defined for desirable outcomes (i.e., higher values are best) as: ${\mathrm{IDP}}_{\text{desirable}}=\frac{\left(E\left(y\right)-x_{\min}\right)}{\left(x_{\max}-x_{\min}\right)}*100\%$ The IDP can then be defined for undesirable outcomes (i.e., lower values are best) as: ${\mathrm{IDP}}_{\text{undesirable}}=100\%-{\mathrm{IDP}}_{\text{desirable}}$

*Note:* Performance metrics automatically calculated by *adaptr*, adapted from a previous article by our group [2]. For the metrics that are not commonly used summary statistics across simulations (means, SDs, quantiles, or proportions), formulae for calculation are provided in the table. Other performance metrics may also be of interest in specific cases, but these are not automatically calculated by *adaptr* and are not covered here.

Abbreviations: IDP: ideal design percentage; IQR: interquartile range (i.e., 25% and 75% percentiles); MAE: median absolute error; RMSE: root mean squared error; SD: standard deviation.

As mentioned previously (Section 2.2), type 1 error rates will typically be of particular importance, especially for late‐phase trials [25, 26] and especially for registrational trials aimed at obtaining marketing authorisations for drugs. For trials with two arms, the type 1 error rate corresponds to the probability of stopping for superiority in the null scenario with no between‐arm differences [2]. For trials with > 2 arms, this is more complex as there is more than a single comparison [66, 67]. The type 1 error rate may be assessed as the probability of superiority in the null scenario with no differences between any arms; this is sometimes referred to as *weakly* controlling the type 1 error rate [66, 67]. However, the probabilities of type 1 error rates, that is, erroneous superiority claims, can also be assessed in other scenarios, including scenarios where some arms are identical, but others are not [66, 67]. The type 1 error rates in such scenarios can be substantially higher than in the null scenario [66] and controlling type 1 error rates across a range of realistic scenarios consisting of all possible combinations of no differences and differences (possibly of different magnitudes, as discussed in Section 3.2.7) should be considered, and will typically be required for trials aimed at obtaining marketing authorisations [66, 67]. As the type 1 error rate cannot be guaranteed for scenarios not evaluated, we recommend that a range of scenarios covering the plausible, realistic differences be assessed.

Some performance metrics (arm selection probabilities, error metrics, and ideal design percentages) are calculated based on the *selected* arms. Different *arm selection strategies* may be chosen for simulations where superiority is not concluded, based on, for example, which arm would be used in clinical practice afterwards if the trial is inconclusive [2]. For example, these performance metrics may be calculated in the following ways:

- For trial simulations ending with superiority only.
- For simulations not ending with superiority:
  ○ Considering the original common control arm (if any and if not dropped previously) selected in simulations not ending with superiority.
  ○ Considering a specific arm (i.e., the cheapest/most available intervention) selected in simulations not ending with superiority.
  ○ Considering the arm with the highest probability of being best at the last analysis selected in simulations not ending for superiority.

Some metrics may be prioritised for logistical/economic reasons (e.g., mean sample sizes), to maximise benefits to *participants* (e.g., total event counts and event probabilities), to maximise benefits to *external* and future patients (probabilities of conclusiveness/superiority, type 1 error rates, power, arm selection probabilities, ideal design percentages [2, 18]), and to maximise the accuracy of the trial results (error metrics). The probabilities of superiority and conclusiveness, type 1 error rates, power, and the expected sample sizes will typically be of high priority.

### 3.4 Simulations and Performance Metric Calculation

Many trial simulations are needed to accurately assess performance and possibly compare multiple candidate designs. Importantly, evaluated performance metrics depend not only on the trial design but also on the assumptions underlying the simulations (which should be challenged, as discussed in Section 3.8) and on adherence to all adaptation rules. If adaptation rules are not binding or if binding rules are not strictly followed, the performance metric estimates will be invalid [26]. Performance may be assessed directly, followed by manually revising and re‐assessing trial designs in an iterative process until performance metrics are acceptable. Alternatively, an automatic process may be used to *calibrate* a specific design parameter to obtain an acceptable value for a specific performance metric (Section 3.3). Typically, ensuring that stopping rules for superiority/inferiority lead to acceptable type 1 error rates is central. Manual assessment may be carried out by conducting simulations under the *primary null* scenario with specified stopping rules and assessing whether the overall type 1 error in this scenario and other metrics are acceptable. If the type 1 error rate is too high in this scenario, stopping thresholds may be made more restrictive, followed by a new round of simulations in an iterative fashion until the overall type 1 error is acceptable. Similarly, if the type 1 error rate is below the desired value, stopping rules may be made more lenient, followed by repeated simulations and evaluation to decrease the expected sample size. Alternatively, this may be done using an automatic calibration procedure (Section 3.5). Other elements of the trial design may also be iteratively revised to ensure that other performance metrics are acceptable. Conducting 100,000 simulations is generally recommended when evaluating type 1 error rates [26], but fewer (e.g., 10,000) simulations may be enough for evaluating other metrics where less precision is required or if uncertainty measures (e.g., 95% confidence intervals [CIs]) are calculated and found acceptable [26].

In *adaptr*, trial simulations may be conducted using *run_trials()*. When simulations have been conducted, results may be calculated, extracted, and summarised using multiple functions. *extract_results()* returns data in a tabular (*data.frame*) format with one row per simulation and one column per data point. *check_performance()* summarises performance metrics across trials in a *data.frame* format with optional calculation of uncertainty measures, for example, 95% CIs, calculated using non‐parametric bootstrapping with resampling with replacement of the results obtained from the individual simulations [68]. Finally, *summary()* calculates performance metrics and summarises simulation results in a *list* format with a dedicated print method.

In the example, we conduct 10,000 simulations using the previously defined trial specification under the *primary null* scenario (Section 3.2.7). This is followed by the calculation of performance metrics with uncertainty measures in the form of 95% CIs, with no arm selected in simulations not stopped for superiority (Section 3.3):


primary_sims_uncalibrated <- **run_trials**( trial_spec = primary_design_null_scenario, n_rep = 10000, base_seed = 4131, *# Reproducibility* path = **paste0**(dir_out, "Primary sims uncalibrated.RDS") *# Save/reload*)primary_performance_uncalibrated <- **check_performance**( primary_sims_uncalibrated, select_strategy = "none", uncertainty = TRUE, n_boot = 5000, *# Number of bootstrap resamples* ci_width = 0.95, *# 95% CIs* boot_seed = 4131 *# Reproducibility*)



All performance metrics are included in Appendix A in Supporting Information; the percentage of simulations stopped for superiority, that is, the type 1 error rate in this scenario, is 5.3% (95% CI: 4.8% to 5.7%), with a mean sample size of 7881 participants. Even though fewer simulations are conducted than the 100,000 recommended, the actual estimate and the 95% CI indicate that the type 1 error rate is likely to exceed the typically recommended 5%. Consequently, to ensure an acceptable type 1 error rate in this scenario, it may be necessary to change the stopping rules (or at least conduct a larger number of simulations to decrease the uncertainty, in which case the type 1 error rate in this scenario could turn out to be acceptable).

### 3.5 Calibration

To adequately control the overall type 1 error rate for the guiding outcome in the null scenario, stopping rules for superiority/inferiority may be automatically calibrated. Calibration according to a specific desired type 1 error rate should be performed under the *primary null scenario* and combines repeated simulations with an algorithm that aims to find stopping thresholds that achieve the desired type 1 error rate.

*adaptr* not only supports automatic calibration of trial specifications to obtain constant, symmetrical stopping rules for superiority/inferiority that target the typically recommended type 1 error rate of 5% [2, 25, 26, 35] but also supports calibration of non‐constant/non‐symmetric stopping rules or other design choices to optimise another performance metric. *adaptr* uses a Gaussian process‐based Bayesian optimisation algorithm [69] that aims to efficiently (i.e., with as few sets of simulations as possible) identify stopping rules that will lead to the desired type 1 error rate. Below, we calibrate the superiority and inferiority stopping rules using *calibrate_trial()* with 10,000 simulations in each calibration step. A *target* value for the type 1 error rate (5%), a tolerance threshold and direction of tolerance, a search range for the stopping threshold for superiority (with the threshold for inferiority defined as 1—the threshold for superiority in this case), along with a maximum number of iterations allowed, determines when the calibration procedure is stopped:primary_design_null_scenario_calibration <- **calibrate_trial**( trial_spec = primary_design_null_scenario, n_rep = 10000, base_seed = 4131, *# Reproducibility# Target, search range, tolerance, and maximum number of iterations* target = 0.05, search_range = **c**(0.9, 1), tol = 0.001, dir = ‐1, *# Only tolerate values below target, i.e., 0.049 to 0.050* iter_max = 25, path = **paste0**(dir_out, "Primary calibration.RDS") *# Save/reload*)


#### 3.5.1 Evaluating the Calibration Procedure and Results

Following the calibration process, it should be checked if the calibration procedure was successful, that is, that an acceptable type 1 error rate was achieved within the maximum permitted number of iterations. If not, consider using more posterior draws, more iterations, a wider search range, or a wider tolerance range for the target value. Example code to summarise calibration results and extract simulations and other data following calibration, including the relevant code outputs, is included in Appendices A and B in Supporting Information.

In the example, the calibration was successful, with a resulting stopping threshold for superiority of 0.990416 (rounded to six significant digits and corresponding to a stopping threshold for inferiority of 0.009584). As only 10,000 simulations were conducted during each iteration in the calibration process, uncertainty measures should be calculated and checked, *or* alternatively, the final calibrated trial design should be evaluated using 100,000 simulations to ensure that the type 1 error rate (and other performance metrics) remains acceptable. For practical purposes, stopping rules with a limited number of digits are easier to use; however, rounding requires a new evaluation. Below, the calibrated stopping rules are rounded to four significant digits, followed by the conduct of 100,000 simulations and performance evaluation:*# Extract and round calibrated stopping rule for superiority (‘best_x’)*superiority_rounded <- **round**(primary_design_null_scenario_calibration**$**best_x, 4)*# Extract calibrated trial design specification (‘best_trial_spec’) and update# to use rounded stopping rules (inferiority = 1‐superiority)*primary_design_null_scenario_calib <‐ primary_design_null_scenario_calibration**$**best_trial_specprimary_design_null_scenario_calib**$**superiority <- superiority_roundedprimary_design_null_scenario_calib**$**inferiority <- 1 ‐ superiority_rounded*# Run large number of simulations with updated trial design specification*primary_null_calibrated <‐ **run_trials**( primary_design_null_scenario_calib, n_rep = 100000, path = **paste0**(dir_out, "Primary sims calibrated.RDS"), *# Save/reload* base_seed = 4131 *# Reproducibility*)*# Check performance metrics without calculating uncertainty measures (not# necessary due to the large number of simulations)*primary_performance_calibrated_rounded <‐ **check_performance**( primary_null_calibrated, select_strategy = "none")


All outputs are included in Appendix A in Supporting Information; the probability of superiority (type 1 error rate) for these 100,000 simulations is 4.8%, with a mean sample size of 7932 participants. As the type 1 error rate in this scenario is still acceptable, we proceed with these stopping rules. Notably, the probability of conclusiveness is only 66.4% due to only 61.6% probability of stopping for practical equivalence, in addition to the 4.8% probability of stopping for superiority. This may be too low, and increasing the maximum sample size or making the stopping rule for practical equivalence more lenient should be considered in cases like this before proceeding with evaluations under other clinical scenarios. For the sake of the example, we, however, proceed with this design and the calibrated stopping rules.

### 3.6 Performance Metric Assessment Under Other Clinical Scenarios

Following successful calibration and if the results are considered acceptable under the *null scenario*, the trial design and calibrated stopping rules may be used to conduct additional simulations evaluating the design under other scenarios as described in Section 3.2.7. Table 2 contains selected performance metrics for the example trial design evaluated under 15 example scenarios constituting the unique combinations of *small* differences, corresponding to the threshold for practical equivalence, and *large* differences, corresponding to two times the equivalence threshold, in both directions (only unique combinations of differences are assessed, as it does not matter here which arm is which due to the lack of a common control arm and the selection strategy used; all performance metrics and the corresponding code are included in Appendices A and B in Supporting Information).

**TABLE 2:** Event probabilities in each arm and selected performance metrics under the 15 clinical scenarios evaluated.

Arm A	Arm B	Arm C	Mean sample size	Probability of conclusiveness^a^	Probability of superiority^b^	Probability of equivalence^c^	Probability of erroneous superiority^d^
25.0%	25.0%	25.0%	7932	66.4%	4.8%	61.6%	4.8%
25.0%	27.5%	25.0%	6496	85.2%	14.6%	70.6%	14.6%
25.0%	22.5%	25.0%	6473	81.5%	59.7%	21.8%	0.4%
25.0%	30.0%	25.0%	5304	97.1%	14.1%	83.0%	14.1%
25.0%	20.0%	25.0%	2871	100.0%	99.6%	0.4%	0.0%
25.0%	27.5%	27.5%	6710	77.5%	56.5%	21.1%	0.5%
25.0%	22.5%	27.5%	5052	95.2%	74.3%	20.9%	0.6%
25.0%	30.0%	27.5%	5287	93.1%	73.0%	20.1%	0.7%
25.0%	20.0%	27.5%	2350	100.0%	99.8%	0.2%	0.0%
25.0%	22.5%	22.5%	6239	87.2%	13.7%	73.6%	13.7%
25.0%	30.0%	22.5%	4716	96.4%	74.6%	21.8%	0.7%
25.0%	20.0%	22.5%	4788	96.7%	75.5%	21.2%	0.6%
25.0%	30.0%	30.0%	3205	99.8%	99.3%	0.5%	0.1%
25.0%	20.0%	30.0%	2211	100.0%	99.8%	0.2%	0.1%
25.0%	20.0%	20.0%	4650	99.0%	13.8%	85.1%	13.8%

*Note:* Event probabilities in each arm and selected performance metrics under the 15 clinical scenarios evaluated. The scenarios have constant assumed event rates of 25.0% in one arm and varying event probabilities and combinations in the other arms, corresponding to the primary *null* scenario and the unique combinations of no/small/large differences in both directions. We conducted 100,000 simulations of the scenario without differences between arms but only 10,000 simulations for each scenario with between‐arm differences, as these are not used for calibrating the stopping rules and as less accuracy for the other performance metrics may often be acceptable than for type 1 error rates. Uncertainty measures for all metrics can be calculated if required and are mostly relevant with < 100,000 simulations (Section 3.4).

^a^ Probability of triggering any stopping rule at or before the maximum allowed sample size, that is, the probability of either superiority or practical equivalence in this case.

^b^ The probability of superiority corresponds to the type 1 error rate in the scenario without differences between arms and the power in all scenarios with differences between arms [2].

^c^ The probability of equivalence refers to the final decision between all arms remaining at the last conducted analysis. In this example, one arm may be dropped for inferiority early, and the remaining two arms may then be declared practically equivalent at a later analysis. Proportions of various combinations of available arms in the last analysis conducted in a set of simulations can be summarised using the *check_remaining_arms()* function in *adaptr* [27].

^d^ The probability of erroneous superiority corresponds to the summed probabilities of ultimately declaring an arm overall superior if it is not the sole superior arm in that scenario (i.e., if two arms are practically equivalent, but superior to a third arm, ultimately stopping for superiority for those arms will also be considered erroneous superiority), and may be interpreted as the type 1 error rate for overall superiority decisions across scenarios. Of note, we focus on these probabilities in the example used here, as there is no common control arm, and in this case, we consider the probabilities of erroneous pairwise superiority decisions between arms that are not ultimately declared superior to be less important. This, however, will vary depending on the context, for example, if the trial design focuses on pairwise comparisons of multiple interventional arms against a common control arm.

The probabilities of conclusiveness across the 15 scenarios ranged from 66.4% to approximately 100%, with mean sample sizes ranging from 2211 to 7932. Of note, the probabilities of erroneous overall superiority conclusions (i.e., of the final trial conclusion being superiority, with an arm that is not the single superior arm declared superior) were < 1% in most scenarios with differences present, but up to 14.6% in the scenarios where one arm was inferior to two other identical (superior) arms. Depending on the context (e.g., trial phase and whether the purpose is to obtain marketing authorisations for new drugs), this may be unacceptably high. If so, additional calibration of the stopping rules may be required. This may be done either by manual re‐calibration or by automatic re‐calibration of the stopping rules in the scenario leading to the highest probability of erroneous superiority conclusions, followed by re‐assessment of these re‐calibrated stopping rules in the remaining scenarios.

### 3.7 Sensitivity Analyses Assessing Design Choices

Following the conduct of simulations for the initial trial design under multiple scenarios, additional design variants or rounds of iteration may be necessary to refine the design or compare the effect of different design choices. These choices include, for example, the number and timing of analyses, the priors used [35], the stopping rules (especially those not calibrated, that is, the practical equivalence stopping rule in the example), the randomisation scheme (i.e., the use of fixed‐ or response‐adaptive randomisation or combinations, and any restrictions used with response‐adaptive randomisation), and the follow‐up duration and data outcome‐lag period for the guiding outcome. In the example (Table 2), it may be necessary to increase the maximum sample size or revise the design (including the stopping rules) to increase the probabilities of conclusive results across all clinical scenarios evaluated. Even when the results from the first set of simulations are considered acceptable, sensitivity analyses varying different key design parameters are recommended to assess their potential influence, as this could potentially further improve performance. To limit the number of required simulations compared to doing simulations for all combinations of different values for the key parameters, assessing design variants will typically be done by varying key parameters one at a time, possibly deciding on using a different design variant as the reference, and then eventually further varying other key parameters one at a time. Importantly, whenever *fixed* design parameters (i.e., those controlled by the trialists) are changed and assessed, the calibration process (if used) should generally be repeated; during this process, we recommend that all *assumed* but essentially uncontrollable parameters (e.g., inclusion rates and outcome distributions) be unchanged, followed by assessment using sensitivity analyses later (Section 3.8). Suggested sensitivity analyses are outlined in Table 3.

**TABLE 3:** Suggested sensitivity analyses.

**Suggested sensitivity analyses of fixed design parameters:**
– Other stopping rules for superiority/inferiority (if relevant; mostly relevant if not calibrated)
– Priors used (particularly if informative priors based on external/previous information are used)
– Rounding of stopping rules for superiority/inferiority (if calibrated)
– Other randomisation schemes (e.g., fixed randomisation, more/less restricted response‐adaptive randomisation, different types or degrees of restriction [limits and/or softening factors])
– Different comparison strategy if relevant (e.g., all‐versus‐all comparison if primary design has a common control arm or vice versa if a relevant common control arm may be specified; only relevant for designs with > 2 arms)
– Different analysis timings (including burn‐in) and/or maximum sample sizes
– Different stopping rules for practical equivalence or futility if used (different probability thresholds and stricter thresholds most relevant, but different thresholds over time may also be relevant; if relevant, different absolute thresholds for practical equivalence/futility)
– Different outcome‐data lag periods (if relevant; i.e., different follow‐up duration and/or different permitted lag period for data collection, cleaning, and verification)
**Suggested sensitivity analyses of assumed parameters:**
– Different reference distributions (differences in both directions, e.g., different reference event probabilities; either a grid of different values or a range of values representing the range of a priori plausible values)
– Different inclusion rates (in both directions, either in a grid or a range of values representing what is a priori considered plausible; possibly different inclusion rates over time, for example, if constant inclusion rates are assumed, challenging this may be considered)

*Note:* Suggested sensitivity analyses for consideration when evaluating an advanced adaptive trial using simulations. For the sensitivity analysis of fixed design parameters, any calibration procedure should be repeated when these are changed (except for the evaluation of rounding calibrated stopping rules). For the sensitivity analyses of assumed parameters, the stopping rules should be identical to the primary evaluation, that is, any calibration procedure should not be repeated here. Sensitivity analyses should be conducted using a set of scenarios corresponding to those used for the primary analyses.

### 3.8 Sensitivity Analyses of Assumed Parameters

For at least the final design, sensitivity analyses should be conducted to assess the potential impact of the *assumed* parameters, while the *fixed* design parameters are kept constant (see Table 3 for suggestions). It is especially important to assess the influence of different assumed reference outcome distributions, as this may affect all performance metrics, including type 1 error rates, power, and expected sample sizes [26]. Importantly, key performance metrics should be acceptable across a range or grid of values covering the plausible, different reference distributions [26]. We also recommend sensitivity analyses varying the assumed inclusion rates [40]. Sensitivity analyses covering the range of plausible values for each parameter should be conducted, and the resulting performance metrics should be acceptable under the full range of reasonably plausible assumptions. It is central that such sensitivity analyses are conducted using the *same* stopping rules as in the corresponding simulations conducted under the primary assumptions (as the stopping rules need to be determined prior to trial start), i.e., if the stopping rules were calibrated under the primary *null scenario*, the exact same stopping rules resulting from that calibration should be used in the sensitivity analyses without recalibration.

### 3.9 Reporting

All design characteristics, assumptions, and performance metrics from both the primary simulations and sensitivity analyses of the final trial design should be reported as part of the trial protocol (or in a simulation appendix). Further, presenting results from sensitivity analyses of design variants and assumed parameters and earlier iterations of the trial design, even if not used, is recommended, as this makes the decision of the final trial design transparent. Examples from actual trial protocols are available elsewhere [52, 70]. Including the simulation code (including random seeds and software version info) when reporting results may be considered to increase transparency and allow replication and serve as an aide for other trialists planning similar designs [71, 72].

### 3.10 Additional Examples

Additional examples, including more customised designs using *setup_trial()*, are included in Appendices C and D in Supporting Information:

– Example 1 illustrates how to use a common control arm.
– Example 2 illustrates how to use a more complex outcome distribution.
– Examples 2 and 3 illustrate how to define a custom function to return posterior draws, which may use any estimation method and any desired priors.

Further examples, including examples explicitly considering inclusion rates and outcome‐data lag in greater detail, are available elsewhere [40, 52, 70, 73].

## 4 Discussion

We have provided a thorough example‐based guide on the steps required for evaluating and comparing Bayesian advanced adaptive trial designs using adaptive stopping, arm dropping, and response‐adaptive randomisation, along with full simulation code using a well‐documented and flexible simulation engine [27].

### 4.1 Strengths and Limitations

The primary strength of this manuscript is that we have covered the key methodological decisions needed when planning Bayesian advanced adaptive trials from a theoretical and practical point of view, including providing complete, annotated code covering the entire workflow. Given that limited guidance on this topic exists, we hope this will serve as a valuable reference for trialists considering or using these designs. The *adaptr* package used [27] has the benefits of being open‐source, freely available, relatively easy to use, well‐documented, extensible, and optimised to be relatively fast. However, as previously stated, other software packages for adaptive trial simulation exist [28, 33] and may also be considered.

This guide and our simulation engine come with some limitations. First, while we have aimed to provide comprehensive guidance on assessing and comparing Bayesian advanced adaptive trials, not every adaptive feature is covered here or supported by the *adaptr* [27] package. Primarily, adaptive enrichment (restricting allocation to those most probable to benefit) [74] including separate adaptations in different subgroups [10], and adaptive arm adding (including *staggered entry*) as used in some adaptive platform trials [10] are not covered or supported by the package. However, this framework supports the planning of platform trials with interventions nested in *domains* (groups of comparable interventions similar to what could be compared in a stand‐alone trial) as long as the platform only allows the addition of new domains (which may include interventions assessed in previous domains), but not new interventions within domains (i.e., domains are *closed*) [1, 2, 10, 14]. Second, while we have provided guidance on assessing and comparing different Bayesian advanced adaptive trial designs, we provide limited guidance on the choice of specific adaptive features beyond that they should be compared in each case using simulation. This is intentional, as some adaptive features may be beneficial in some situations while having undesirable effects in other situations; this balance also depends on the prioritisation of different performance metrics (e.g., response‐adaptive randomisation may lower expected sample sizes in some trial designs and increase them in others [2], while increasing individual participants' chances of better outcomes in both scenarios). While some general intuition is provided, it is important not to blindly rely on this or previous results but to evaluate key design choices specifically in each case. Further, it is not possible to provide universal guidance on the prioritisation of different performance metrics, as the prioritisation and acceptable trade‐offs need to be considered separately in each trial being planned. Third, limiting the scope somewhat was necessary, and consequently, we primarily focused on late‐phase, large, and pragmatic Bayesian adaptive trials. As such, adaptive trials using frequentist statistical methods or adaptive trial designs used for earlier phase trials (including dose‐finding trials) are not covered here. However, most of the considerations discussed also apply to such trial designs, even if the planning, evaluating, and final interpretation may differ somewhat. An intentional limitation is thus that no direct comparisons with, e.g., frequentist adaptive designs or fixed designs are made. While such comparisons are beyond the scope of the present manuscript, they can be very relevant, and comparisons of Bayesian advanced adaptive trial designs, as covered here, with other trial designs should be considered in practice. Fourth, the main text has only covered a design with no common control arm and using a binary outcome with default, flat priors; however, the *adaptr* package documentation and Appendices C and D in Supporting Information provide examples on how to specify simulations using other outcome types, custom priors, common control arms, and other of the more complex features of *adaptr* [27] and the rest of the workflow is similar.

### 4.2 Conclusions

In conclusion, this practical guide provides comprehensive advice for trialists considering or planning Bayesian advanced adaptive trials using adaptive stopping, arm dropping, and response‐adaptive randomisation. By including examples of simulation‐based trial design assessment and comparison, we have covered not only the methodological considerations but also the practical aspects of doing simulation‐based trial design evaluation and comparison. While planning and simulating advanced adaptive trials is an iterative process that typically will be more time‐consuming than designing and planning conventional trials, the additional effort in the planning phase will often be outweighed by higher flexibility, increased effectiveness, and higher probabilities of conclusiveness in the resulting trials.

## Author Contributions

Conceptualisation: A.G., A.K.G.J., B.S.K.‐H. Data curation: A.G. Formal analysis: A.G. Funding acquisition: A.G., T.L., A.P., M.H.M. Investigation: A.G. Methodology: all authors. Project administration: A.G. Software: A.G., A.K.G.J., T.L., B.S.K.‐H. Visualisation: A.G. Writing – original draft: A.G. Writing – review and editing: all authors.

## Conflicts of Interest

The authors declare no conflicts of interest.

## Supporting information


**Appendix A.** The complete, annotated simulation code used for the primary example is included in Appendices A and B (Appendix A is a formatted PDF including code, explanation, and all results). All outputs are included in Appendix A.

**Appendix B.** R script only containing code and explanation.

**Appendix C.** Descriptions of additional examples and the corresponding code are included in Appendices C and D (Appendix C is a formatted PDF including code, explanation, and outputs/figures).

**Appendix D.** R script only containing code and explanation.

## Data Availability

This study is based on simulated data only. The complete, annotated analysis code used to generate the simulated data is available in the Supporting Information.
