# Supplementary material for: Designing and Evaluating Bayesian Advanced Adaptive Randomised Clinical Trials: A Practical Guide
Source: Pharm Stat. 2025 Oct 9;24(6):e70042. doi: 10.1002/pst.70042 (PMC12509790; doi:10.1002/pst.70042)
Supplement: Supplementary file 1 — Appendix A. The complete, annotated simulation code used for the primary example is included in Appendices A and B (Appendix A is a formatted PDF including code, explanation, and all results). All outputs are included in Appendix A. [file PST-24-0-s002.pdf]

Designing and evaluating Bayesian advanced adaptive randomised clinical trials: a practical guide

Appendix A

Anders Granholm[1,2], Aksel Karl Georg Jensen[1,2], Theis Lange[2],
Anders Perner[1,3], Morten Hylander Møller[1,3], and Benjamin Skov Kaas-Hansen[1,2]

[1] Department of Intensive Care 4131, Copenhagen University Hospital – Rigshospitalet, Copenhagen, Denmark
[2] Section of Biostatistics, Department of Public Health, University of Copenhagen, Copenhagen, Denmark
[3] Department of Clinical Medicine, Faculty of Health and Medical Sciences, University of Copenhagen, Copenhagen, Denmark

Correspondence: Anders Granholm (anders.granholm@regionh.dk)

Appendix A

This supplementary appendix includes the complete simulation code used for the primary example in the manuscript along with the outputs. **Appendix B** essentially contains the same code, but as a raw R script with explanation in comments and without outputs.

For additional information on using the ***adaptr*** package and on the package outputs, please see the complete package documentation available at: https://inceptdk.github.io/adaptr/.

Setup

Below, the package is loaded and a cluster for parallel computation initiated (the number of cores used may be changed by the user as needed and considering the number of cores available). In addition, the directory used to save simulations and results in is specified (**note: an actual path must be inserted here**). This is done to avoid having to re-run simulations when no changes have been made.

```
library(adaptr)

## Loading 'adaptr' package v1.4.0.
## For instructions, type 'help("adaptr")'
## or see https://inceptdk.github.io/adaptr/.

setup_cluster(10) # Number of cores for parallel computation
dir_out <- "<PATH>/" # Replace with an actual, permanent path
```

Code snippets

The following section includes all code snippets included in the main text of the manuscript as part of the setup of the trial specification (under subheadings corresponding to those in the main text).

These snippets are incomplete and hence not run before the full trial specification is provided below.

Interventions and use of common control

```
setup_trial_binom(
  arms = c("Arm A", "Arm B", "Arm C"),
  control = NULL,
  ...
)
```

Outcome type and generation

```
setup_trial_binom(
  ...
  true_ys = c(0.25, 0.25, 0.25),
  highest_is_best = FALSE,
  ...
)
```

Analysis timing and outcome-data lag

```
setup_trial_binom(
  …
  # Number of participants with data available and included in each analysis
  data_looks = seq(from = 500, to = 10000, by = 250),
  # Number of participants randomised at each analysis
  randomised_at_looks = c(seq(from = 700, to = 9950, by = 250), 10000),
  # Note: the maximum number in both arguments should be equal
  …
)
```

Allocation profiles

```
setup_trial_binom(
  ...
  start_probs = c(1/3, 1/3, 1/3),
  fixed_probs = NULL,
  min_probs = c(0.25, 0.25, 0.25),
  rescale_probs = "limits",
  soften_power = 0.5,
  ...
)
```

Analysis model and priors

```
setup_trial_binom(
  …
  n_draws = 10000,
  …
)
```

Stopping and arm dropping rules

```
setup_trial_binom(
  ...
  inferiority = 0.01,
  superiority = 0.99,
  equivalence_prob = ifelse(seq(from = 500, to = 10000, by = 250) < 1500, 1, 0.9),
  equivalence_diff = 0.025,
  ...
)
```

Complete trial design specification

Here, all the incomplete snippets are combined into a complete trial specification (with fewer comments), which can be run:

```
# Specification
primary_design_null_scenario <- setup_trial_binom(
  # Arms and scenario
  arms = c("Arm A", "Arm B", "Arm C"),
  control = NULL,
  true_ys = c(0.25, 0.25, 0.25),
  highest_is_best = FALSE,
  # Allocation rules
  start_probs = c(1/3, 1/3, 1/3),
  fixed_probs = NULL,
  min_probs = c(0.25, 0.25, 0.25),
  rescale_probs = "limits",
  soften_power = 0.5,
  # Participants with data available/randomised at each analysis
  data_looks = seq(from = 500, to = 10000, by = 250),
  randomised_at_looks = c(seq(from = 700, to = 9950, by = 250), 10000),
  # Stopping rules
  inferiority = 0.01,
  superiority = 0.99,
  equivalence_prob = ifelse(seq(from = 500, to = 10000, by = 250) < 1500, 1, 0.9),
  equivalence_diff = 0.025,
  # Posterior draws
  n_draws = 10000
)

# Print design specification
primary_design_null_scenario

## Trial specification: generic binomially distributed outcome trial
## * Undesirable outcome
## * No common control arm
## * Best arms: Arm A and Arm B and Arm C
##
## Arms, true outcomes, starting allocation probabilities
## and allocation probability limits (min/max_probs rescaled):
##   arms true_ys start_probs fixed_probs min_probs max_probs
##  Arm A    0.25       0.333          NA      0.25        NA
```

```
##   Arm B    0.25       0.333          NA      0.25        NA
##   Arm C    0.25       0.333          NA      0.25        NA
##
## Maximum sample size: 10000
## Maximum number of data looks: 39
## Planned data looks after:  500, 750, 1000, 1250, 1500, 1750, 2000, 2250, 2500, 2750,
3000, 3250, 3500, 3750, 4000, 4250, 4500, 4750, 5000, 5250, 5500, 5750, 6000, 6250, 6500,
6750, 7000, 7250, 7500, 7750, 8000, 8250, 8500, 8750, 9000, 9250, 9500, 9750, 10000
patients have reached follow-up
## Number of patients randomised at each look:  700, 950, 1200, 1450, 1700, 1950, 2200,
2450, 2700, 2950, 3200, 3450, 3700, 3950, 4200, 4450, 4700, 4950, 5200, 5450, 5700, 5950,
6200, 6450, 6700, 6950, 7200, 7450, 7700, 7950, 8200, 8450, 8700, 8950, 9200, 9450, 9700,
9950, 10000
##
## Superiority threshold: 0.99 (all analyses)
## Inferiority threshold: 0.01 (all analyses)
## Equivalence thresholds:
## 1, 1, 1, 1, 0.9, 0.9, 0.9, 0.9, 0.9, 0.9, 0.9, 0.9, 0.9, 0.9, 0.9, 0.9, 0.9, 0.9, 0.9,
0.9, 0.9, 0.9, 0.9, 0.9, 0.9, 0.9, 0.9, 0.9, 0.9, 0.9, 0.9, 0.9, 0.9, 0.9, 0.9, 0.9, 0.9,
0.9, 0.9
## (no common control)
## Absolute equivalence difference: 0.025
## No futility threshold (not relevant - no common control)
## Soften power for all analyses: 0.5
```

Simulations and performance metric evaluation without calibration

```
primary_sims_uncalibrated <- run_trials(
  trial_spec = primary_design_null_scenario,
  n_rep = 10000,
  base_seed = 4131, # Reproducibility
  path = paste0(dir_out, “Primary sims uncalibrated.RDS”) # Save/reload
)

primary_performance_uncalibrated <- check_performance(
  primary_sims_uncalibrated,
  select_strategy = “none”,
  uncertainty = TRUE,
  n_boot = 5000, # Number of bootstrap resamples
  ci_width = 0.95, # 95% CIs
  boot_seed = 4131 # Reproducibility
)

# Print and save
primary_performance_uncalibrated

##                  metric       est   err_sd err_mad     lo_ci     hi_ci
## 1          n_summarised 10000.000    0.000   0.000 10000.000 10000.000
## 2             size_mean  7880.700   24.402  23.900  7831.789  7928.992
## 3               size_sd  2436.432   14.790  14.670  2407.666  2465.283
## 4           size_median  8950.000    6.122   0.000  8950.000  8950.000
## 5              size_p25  5450.000 119.514   0.000  5450.000  5700.000
```

```
## 6              size_p75 10000.000   0.000   0.000 10000.000 10000.000
## 7               size_p0   700.000      NA      NA        NA        NA
## 8              size_p100 10000.000     NA      NA        NA        NA
## 9            sum_ys_mean  1969.661   6.105   5.992  1957.485  1981.875
## 10             sum_ys_sd   610.416   3.708   3.695   603.144   617.657
## 11         sum_ys_median  2230.000   8.779   8.896  2215.500  2249.000
## 12            sum_ys_p25  1394.000  14.147  11.305  1367.000  1425.000
## 13            sum_ys_p75  2484.000   1.169   1.483  2482.000  2486.000
## 14             sum_ys_p0   157.000      NA      NA        NA        NA
## 15           sum_ys_p100  2656.000      NA      NA        NA        NA
## 16         ratio_ys_mean     0.250   0.000   0.000     0.250     0.250
## 17           ratio_ys_sd     0.005   0.000   0.000     0.005     0.005
## 18       ratio_ys_median     0.250   0.000   0.000     0.250     0.250
## 19          ratio_ys_p25     0.247   0.000   0.000     0.246     0.247
## 20          ratio_ys_p75     0.253   0.000   0.000     0.253     0.253
## 21           ratio_ys_p0     0.218      NA      NA        NA        NA
## 22         ratio_ys_p100     0.306      NA      NA        NA        NA
## 23       prob_conclusive     0.669   0.005   0.005     0.659     0.678
## 24         prob_superior     0.053   0.002   0.002     0.048     0.057
## 25      prob_equivalence     0.616   0.005   0.005     0.607     0.626
## 26         prob_futility     0.000   0.000   0.000     0.000     0.000
## 27              prob_max     0.331   0.005   0.005     0.322     0.340
## 28 prob_select_arm_Arm A     0.016   0.001   0.001     0.014     0.019
## 29 prob_select_arm_Arm B     0.017   0.001   0.001     0.015     0.020
## 30 prob_select_arm_Arm C     0.019   0.001   0.001     0.016     0.022
## 31      prob_select_none     0.947   0.002   0.002     0.943     0.952
## 32                  rmse     0.032   0.001   0.001     0.030     0.034
## 33               rmse_te        NA      NA      NA        NA        NA
## 34                   mae     0.022   0.001   0.001     0.020     0.024
## 35                mae_te        NA      NA      NA        NA        NA
## 36                   idp        NA      NA      NA        NA        NA

write.csv2(
  primary_performance_uncalibrated,
  file = paste0(dir_out, “Performance primary sims uncalibrated.csv”),
  row.names = FALSE
)
```

Calibration

```
primary_design_null_scenario_calibration <- calibrate_trial(
  trial_spec = primary_design_null_scenario,
  n_rep = 10000,
  base_seed = 4131, # Reproducibility
  # Target, search range, tolerance, and maximum number of iterations
  target = 0.05,
  search_range = c(0.9, 1),
  tol = 0.001,
  dir = -1, # Only tolerate values below target, i.e., 0.049 to 0.050
  iter_max = 25,
  path = paste0(dir_out, "Primary calibration.RDS") # Save/reload
)
```

```
# Print summary of calibration results
primary_design_null_scenario_calibration

## Trial calibration:
## * Result: calibration successful
## * Best x: 0.9904165
## * Best y: 0.0492
##
## Central settings:
## * Target: 0.05
## * Tolerance: 0.001 (at or below target, range: 0.049 to 0.05)
## * Search range: 0.9 to 1
## * Gaussian process controls:
## * - resolution: 5000
## * - kappa: 0.5
## * - pow: 1.95
## * - lengthscale: 1 (constant)
## * - x scaled: yes
## * Noisy: no
## * Narrowing: yes
##
## Calibration/simulation details:
## * Total evaluations: 7 (previous + grid + iterations)
## * Repetitions: 10000
## * Calibration time: 1.22 hours
## * Base random seed: 4131
##
## See 'help("calibrate_trial")' for details.

# Check if successful (should be TRUE)
primary_design_null_scenario_calibration$success

## [1] TRUE

# Extract simulations conducted using the best stopping rule
primary_design_null_scenario_calibration$best_sims

## Multiple simulation results: generic binomially distributed outcome trial
## * Undesirable outcome
## * Number of simulations: 10000
## * Number of simulations summarised: 10000 (all trials)
## * No common control arm
## * Selection strategy: no selection if no superior arm
## * Treatment effect compared to: no comparison
##
## Performance metrics (using posterior estimates from final analysis [all patients]):
## * Sample sizes: mean 7935.0 (SD: 2414.4) | median 8950.0 (IQR: 5700.0 to 10000.0)
[range: 700.0 to 10000.0]
## * Total summarised outcomes: mean 1983.2 (SD: 604.9) | median 2248.0 (IQR: 1424.0 to
2486.0) [range: 157.0 to 2656.0]
## * Total summarised outcome rates: mean 0.250 (SD: 0.005) | median 0.250 (IQR: 0.247 to
0.253) [range: 0.218 to 0.306]
## * Conclusive: 66.1%
```

```
## * Superiority: 4.9%
## * Equivalence: 61.2%
## * Futility: 0.0% [not assessed]
## * Inconclusive at max sample size: 33.9%
## * Selection probabilities: Arm A: 1.5% | Arm B: 1.6% | Arm C: 1.8% | None: 95.1%
## * RMSE / MAE: 0.03233 / 0.02218
## * RMSE / MAE treatment effect: not estimated / not estimated
## * Ideal design percentage: not estimable
##
## Simulation details:
## * Simulation time: 15 mins
## * Base random seed: 4131
## * Credible interval width: 95%
## * Number of posterior draws: 10000
## * Estimation method: posterior medians with MAD-SDs
```

```
# Extract calibrated trial specification
primary_design_null_scenario_calibration$best_trial_spec
```

```
## Trial specification: generic binomially distributed outcome trial
## * Undesirable outcome
## * No common control arm
## * Best arms: Arm A and Arm B and Arm C
##
## Arms, true outcomes, starting allocation probabilities
## and allocation probability limits (min/max_probs rescaled):
##   arms true_ys start_probs fixed_probs min_probs max_probs
##  Arm A    0.25       0.333          NA      0.25        NA
##  Arm B    0.25       0.333          NA      0.25        NA
##  Arm C    0.25       0.333          NA      0.25        NA
##
## Maximum sample size: 10000
## Maximum number of data looks: 39
## Planned data looks after:  500, 750, 1000, 1250, 1500, 1750, 2000, 2250, 2500, 2750,
3000, 3250, 3500, 3750, 4000, 4250, 4500, 4750, 5000, 5250, 5500, 5750, 6000, 6250, 6500,
6750, 7000, 7250, 7500, 7750, 8000, 8250, 8500, 8750, 9000, 9250, 9500, 9750, 10000
patients have reached follow-up
## Number of patients randomised at each look:  700, 950, 1200, 1450, 1700, 1950, 2200,
2450, 2700, 2950, 3200, 3450, 3700, 3950, 4200, 4450, 4700, 4950, 5200, 5450, 5700, 5950,
6200, 6450, 6700, 6950, 7200, 7450, 7700, 7950, 8200, 8450, 8700, 8950, 9200, 9450, 9700,
9950, 10000
##
## Superiority threshold: 0.99042 (all analyses)
## Inferiority threshold: 0.00958 (all analyses)
## Equivalence thresholds:
## 1, 1, 1, 1, 0.9, 0.9, 0.9, 0.9, 0.9, 0.9, 0.9, 0.9, 0.9, 0.9, 0.9, 0.9, 0.9, 0.9, 0.9,
0.9, 0.9, 0.9, 0.9, 0.9, 0.9, 0.9, 0.9, 0.9, 0.9, 0.9, 0.9, 0.9, 0.9, 0.9, 0.9, 0.9, 0.9,
0.9, 0.9
## (no common control)
## Absolute equivalence difference: 0.025
## No futility threshold (not relevant - no common control)
## Soften power for all analyses: 0.5
```

```
# Extract stopping threshold for superiority (using the default functionality
# the stopping threshold for inferiority is 1 - this value)
primary_design_null_scenario_calibration$best_x

## [1] 0.9904165
```

Round stopping rules after calibration and re-assess

```
# Extract and round calibrated stopping rule for superiority ('best_x')
superiority_rounded <- round(primary_design_null_scenario_calibration$best_x, 4)

# Extract calibrated trial design specification ('best_trial_spec') and update
# to use rounded stopping rules (inferiority = 1 - superiority)
primary_design_null_scenario_calib <-
  primary_design_null_scenario_calibration$best_trial_spec
primary_design_null_scenario_calib$superiority <- superiority_rounded
primary_design_null_scenario_calib$inferiority <- 1 - superiority_rounded

# Run large number of simulations with updated trial design specification
primary_null_calibrated <- run_trials(
  primary_design_null_scenario_calib,
  n_rep = 100000,
  path = paste0(dir_out, "Primary sims calibrated.RDS"), # Save/reload
  base_seed = 4131 # Reproducibility
)

# Check performance metrics without calculating uncertainty measures (not
# necessary due to the large number of simulations)
primary_performance_calibrated_rounded <- check_performance(
  primary_null_calibrated,
  select_strategy = "none"
)

# Print and save
primary_performance_calibrated_rounded

##                   metric        est
## 1           n_summarised 100000.000
## 2              size_mean   7931.986
## 3                size_sd   2399.509
## 4            size_median   8950.000
## 5               size_p25   5700.000
## 6               size_p75  10000.000
## 7                size_p0    700.000
## 8              size_p100  10000.000
## 9            sum_ys_mean   1983.076
## 10             sum_ys_sd    601.851
## 11         sum_ys_median   2246.000
## 12            sum_ys_p25   1429.000
## 13            sum_ys_p75   2485.000
## 14             sum_ys_p0    141.000
## 15           sum_ys_p100   2684.000
```

```
## 16          ratio_ys_mean      0.250
## 17            ratio_ys_sd      0.005
## 18        ratio_ys_median      0.250
## 19           ratio_ys_p25      0.247
## 20           ratio_ys_p75      0.253
## 21            ratio_ys_p0      0.201
## 22          ratio_ys_p100      0.306
## 23        prob_conclusive      0.664
## 24          prob_superior      0.048
## 25       prob_equivalence      0.616
## 26          prob_futility      0.000
## 27               prob_max      0.336
## 28 prob_select_arm_Arm A      0.016
## 29 prob_select_arm_Arm B      0.016
## 30 prob_select_arm_Arm C      0.016
## 31       prob_select_none      0.952
## 32                   rmse      0.033
## 33                rmse_te         NA
## 34                    mae      0.022
## 35                 mae_te         NA
## 36                    idp         NA

write.csv2(
  primary_performance_calibrated_rounded,
  file = paste0(dir_out, "Performance primary sims calibrated and rounded.csv"),
  row.names = FALSE
)
```

Performance assessment under other clinical scenarios

First, all relevant scenarios (combinations of effects) are defined. The event probabilities are identical in arm A across all scenarios, but the unique combinations of small and large effects in both directions are specified for the other two arms. Of note, for simulation purposes, it does not matter which arm is which when there is no common control arm, except if using an arm selection strategy for performance metric calculation for simulations not ending with superiority where a specific arm (or a specific order of arms from a supplied list) is chosen. As no arm is selected when calculating performance metrics in this example, duplicate 'identical' combinations of event probabilities across arms B and C are not necessary and removed in the code below:

```
# Possible combinations considered, 3 arms, 3 effect sizes
# Always constant event probabilities in arm A, only unique combinations of
# event probabilities in arms B and C used
effects <- c(0, 0.025, -0.025, 0.05, -0.05)
scenarios <- expand.grid(B = effects, C = effects)
scenarios$A <- 0
scenarios <- scenarios[, c("A", "B", "C")] # Reorder

# Remove non-unique combinations
for (i in nrow(scenarios):2) {
  cur_B <- scenarios[i, "B"]
  cur_C <- scenarios[i, "C"]
  remove <- FALSE
```

```
  for (j in (i-1):2) {
    if (scenarios[j, "B"] == cur_C & scenarios[j, "C"] == cur_B) {
      remove <- TRUE
    }
  }
  if (remove) {
    scenarios <- scenarios[-i, ]
  }
}
rownames(scenarios) <- 1:nrow(scenarios)

# Function for rounding and formatting results
rnd_fmt <- function(x, n = 0, mult = 1, suffix = "", na = "-") {
  res <- paste0(format(round(x * mult, digits = n), nsmall = n), suffix)
  if (!is.null(na)) {
    res <- ifelse(is.na(x), na, res)
  }
  res
}

# Prepare data.frame for formatted key results
key_results <- data.frame(
  A = paste0(rnd_fmt(scenarios$A + 0.25, 1, 100, "%")),
  B = paste0(rnd_fmt(scenarios$B + 0.25, 1, 100, "%")),
  C = paste0(rnd_fmt(scenarios$C + 0.25, 1, 100, "%")),
  size = "",
  pr_concl = NA,
  pr_sup = NA,
  pr_equi = NA,
  pr_err_sup = NA
)

# Run 10,000 simulations for each new scenario (re-use previously calculated
# results for the null scenario), output results for each scenario, and extract
# key results
for (i in 1:nrow(scenarios)) {
  # Current scenario settings
  cur_scenario_name <- paste0(
    "A 25.0 - B ", format(25 + scenarios$B[i] * 100, nsmall = 1),
    " - C ", format(25 + scenarios$C[i] * 100, nsmall = 1))
  cur_true_ys <- c(0.25, 0.25 + scenarios$B[i], 0.25 + scenarios$C[i])
  if (all(cur_true_ys == 0.25)) { # Re-use null scenario results
    cur_sims <- primary_null_calibrated
  } else {
    # Specify trial design with 'new' event probabilities and otherwise same settings
    cur_spec <- setup_trial_binom(
      arms = c("Arm A", "Arm B", "Arm C"),
      control = NULL,
      true_ys = cur_true_ys,
      highest_is_best = FALSE,
      start_probs = c(1/3, 1/3, 1/3),
      fixed_probs = NULL,
```

```
      min_probs = c(0.25, 0.25, 0.25),
      rescale_probs = "limits",
      soften_power = 0.5,
      data_looks = seq(from = 500, to = 10000, by = 250),
      randomised_at_looks = c(seq(from = 700, to = 9950, by = 250), 10000),
      inferiority = 1 - superiority_rounded,
      superiority = superiority_rounded,
      equivalence_prob = ifelse(seq(from = 500, to = 10000, by = 250) < 1500, 1, 0.9),
      equivalence_diff = 0.025,
      n_draws = 10000
    )
    # Run simulations
    cur_sims <- run_trials(
      cur_spec,
      n_rep = 10000,
      path = paste0(dir_out, "Primary ", cur_scenario_name, ".RDS"), # Save/reload
      base_seed = 4131 + i # Reproducibility
    )
  }
  # Summarise results in list format (without uncertainty measures) and print
  cur_res <- summary(cur_sims, select_strategy = "none")
  cat("\n\n##################################################",
      "\nPerformance metrics for scenario:", cur_scenario_name, "\n")
  print(cur_res)
  # Extract and save key results
  key_results$size[i] <- rnd_fmt(cur_res$size_mean)
  key_results$pr_concl[i] <- rnd_fmt(cur_res$prob_conclusive, 1, 100, "%")
  key_results$pr_sup[i] <- rnd_fmt(cur_res$prob_superior, 1, 100, "%")
  key_results$pr_equi[i] <- rnd_fmt(cur_res$prob_equivalence, 1, 100, "%")
  # Erroneous overall superiority conclusions
  best_arms <- names(scenarios)[scenarios[i, ] == min(scenarios[i, ])]
  if (length(best_arms) == 1) { # A single best arm
    err_arms <- names(scenarios)[scenarios[i, ] > min(scenarios[i, ])]
  } else { # Not a single best arm - all erroneous
    err_arms <- names(scenarios)
  }
  key_results$pr_err_sup[i] <- rnd_fmt(
    sum(
      vapply(err_arms,
             \(a) cur_res[[paste0("prob_select_arm_Arm ", a)]], numeric(1))
    ),
    1, 100, "%"
  )
}

## 
## 
## ##################################################
## Performance metrics for scenario: A 25.0 - B 25.0 - C 25.0
## Multiple simulation results: generic binomially distributed outcome trial
## * Undesirable outcome
## * Number of simulations: 1e+05
## * Number of simulations summarised: 1e+05 (all trials)
```

```
## * No common control arm
## * Selection strategy: no selection if no superior arm
## * Treatment effect compared to: no comparison
##
## Performance metrics (using posterior estimates from final analysis [all patients]):
## * Sample sizes: mean 7932.0 (SD: 2399.5) | median 8950.0 (IQR: 5700.0 to 10000.0)
[range: 700.0 to 10000.0]
## * Total summarised outcomes: mean 1983.1 (SD: 601.9) | median 2246.0 (IQR: 1429.0 to
2485.0) [range: 141.0 to 2684.0]
## * Total summarised outcome rates: mean 0.250 (SD: 0.005) | median 0.250 (IQR: 0.247 to
0.253) [range: 0.201 to 0.306]
## * Conclusive: 66.4%
## * Superiority: 4.8%
## * Equivalence: 61.6%
## * Futility: 0.0% [not assessed]
## * Inconclusive at max sample size: 33.6%
## * Selection probabilities: Arm A: 1.6% | Arm B: 1.6% | Arm C: 1.6% | None: 95.2%
## * RMSE / MAE: 0.03258 / 0.02248
## * RMSE / MAE treatment effect: not estimated / not estimated
## * Ideal design percentage: not estimable
##
## Simulation details:
## * Simulation time: 3.14 hours
## * Base random seed: 4131
## * Credible interval width: 95%
## * Number of posterior draws: 10000
## * Estimation method: posterior medians with MAD-SDs
##
##
## #####################################################
## Performance metrics for scenario: A 25.0 - B 27.5 - C 25.0
## Multiple simulation results: generic binomially distributed outcome trial
## * Undesirable outcome
## * Number of simulations: 10000
## * Number of simulations summarised: 10000 (all trials)
## * No common control arm
## * Selection strategy: no selection if no superior arm
## * Treatment effect compared to: no comparison
##
## Performance metrics (using posterior estimates from final analysis [all patients]):
## * Sample sizes: mean 6495.9 (SD: 2389.3) | median 6200.0 (IQR: 4700.0 to 8700.0)
[range: 700.0 to 10000.0]
## * Total summarised outcomes: mean 1656.2 (SD: 615.9) | median 1557.0 (IQR: 1181.0 to
2217.2) [range: 145.0 to 2723.0]
## * Total summarised outcome rates: mean 0.255 (SD: 0.007) | median 0.255 (IQR: 0.251 to
0.259) [range: 0.207 to 0.293]
## * Conclusive: 85.2%
## * Superiority: 14.6%
## * Equivalence: 70.6%
## * Futility: 0.0% [not assessed]
## * Inconclusive at max sample size: 14.8%
## * Selection probabilities: Arm A: 7.3% | Arm B: 0.0% | Arm C: 7.2% | None: 85.4%
## * RMSE / MAE: 0.02356 / 0.01333
```

```
## * RMSE / MAE treatment effect: not estimated / not estimated
## * Ideal design percentage: 99.7%
##
## Simulation details:
## * Simulation time: 5.21 mins
## * Base random seed: 4133
## * Credible interval width: 95%
## * Number of posterior draws: 10000
## * Estimation method: posterior medians with MAD-SDs
##
##
## ##################################################
## Performance metrics for scenario: A 25.0 - B 22.5 - C 25.0
## Multiple simulation results: generic binomially distributed outcome trial
## * Undesirable outcome
## * Number of simulations: 10000
## * Number of simulations summarised: 10000 (all trials)
## * No common control arm
## * Selection strategy: no selection if no superior arm
## * Treatment effect compared to: no comparison
##
## Performance metrics (using posterior estimates from final analysis [all patients]):
## * Sample sizes: mean 6473.4 (SD: 2779.0) | median 6450.0 (IQR: 4200.0 to 9450.0)
[range: 700.0 to 10000.0]
## * Total summarised outcomes: mean 1539.8 (SD: 662.5) | median 1529.0 (IQR: 1015.0 to
2229.0) [range: 144.0 to 2564.0]
## * Total summarised outcome rates: mean 0.238 (SD: 0.007) | median 0.238 (IQR: 0.234 to
0.242) [range: 0.198 to 0.283]
## * Conclusive: 81.5%
## * Superiority: 59.7%
## * Equivalence: 21.8%
## * Futility: 0.0% [not assessed]
## * Inconclusive at max sample size: 18.5%
## * Selection probabilities: Arm A: 0.2% | Arm B: 59.3% | Arm C: 0.2% | None: 40.3%
## * RMSE / MAE: 0.01394 / 0.00602
## * RMSE / MAE treatment effect: not estimated / not estimated
## * Ideal design percentage: 99.3%
##
## Simulation details:
## * Simulation time: 9.23 mins
## * Base random seed: 4134
## * Credible interval width: 95%
## * Number of posterior draws: 10000
## * Estimation method: posterior medians with MAD-SDs
##
##
## ##################################################
## Performance metrics for scenario: A 25.0 - B 30.0 - C 25.0
## Multiple simulation results: generic binomially distributed outcome trial
## * Undesirable outcome
## * Number of simulations: 10000
## * Number of simulations summarised: 10000 (all trials)
## * No common control arm
```

```
## * Selection strategy: no selection if no superior arm
## * Treatment effect compared to: no comparison
##
## Performance metrics (using posterior estimates from final analysis [all patients]):
## * Sample sizes: mean 5304.2 (SD: 1936.0) | median 4950.0 (IQR: 4200.0 to 6200.0)
[range: 700.0 to 10000.0]
## * Total summarised outcomes: mean 1353.0 (SD: 491.1) | median 1257.0 (IQR: 1071.0 to
1596.0) [range: 157.0 to 2675.0]
## * Total summarised outcome rates: mean 0.256 (SD: 0.008) | median 0.255 (IQR: 0.251 to
0.260) [range: 0.224 to 0.310]
## * Conclusive: 97.1%
## * Superiority: 14.1%
## * Equivalence: 83.0%
## * Futility: 0.0% [not assessed]
## * Inconclusive at max sample size: 2.9%
## * Selection probabilities: Arm A: 6.6% | Arm B: 0.0% | Arm C: 7.5% | None: 86.0%
## * RMSE / MAE: 0.02529 / 0.01531
## * RMSE / MAE treatment effect: not estimated / not estimated
## * Ideal design percentage: 100.0%
##
## Simulation details:
## * Simulation time: 7.05 mins
## * Base random seed: 4135
## * Credible interval width: 95%
## * Number of posterior draws: 10000
## * Estimation method: posterior medians with MAD-SDs
##
##
## ##################################################
## Performance metrics for scenario: A 25.0 - B 20.0 - C 25.0
## Multiple simulation results: generic binomially distributed outcome trial
## * Undesirable outcome
## * Number of simulations: 10000
## * Number of simulations summarised: 10000 (all trials)
## * No common control arm
## * Selection strategy: no selection if no superior arm
## * Treatment effect compared to: no comparison
##
## Performance metrics (using posterior estimates from final analysis [all patients]):
## * Sample sizes: mean 2870.7 (SD: 1501.5) | median 2700.0 (IQR: 1700.0 to 3700.0)
[range: 700.0 to 10000.0]
## * Total summarised outcomes: mean 646.9 (SD: 336.0) | median 590.0 (IQR: 390.0 to
843.0) [range: 134.0 to 2304.0]
## * Total summarised outcome rates: mean 0.226 (SD: 0.010) | median 0.226 (IQR: 0.220 to
0.232) [range: 0.188 to 0.294]
## * Conclusive: 100.0%
## * Superiority: 99.6%
## * Equivalence: 0.4%
## * Futility: 0.0% [not assessed]
## * Inconclusive at max sample size: 0.0%
## * Selection probabilities: Arm A: 0.0% | Arm B: 99.6% | Arm C: 0.0% | None: 0.4%
## * RMSE / MAE: 0.01446 / 0.00753
## * RMSE / MAE treatment effect: not estimated / not estimated
```

```
## * Ideal design percentage: 100.0%
##
## Simulation details:
## * Simulation time: 4.07 mins
## * Base random seed: 4136
## * Credible interval width: 95%
## * Number of posterior draws: 10000
## * Estimation method: posterior medians with MAD-SDs
##
##
## ##################################################
## Performance metrics for scenario: A 25.0 - B 27.5 - C 27.5
## Multiple simulation results: generic binomially distributed outcome trial
## * Undesirable outcome
## * Number of simulations: 10000
## * Number of simulations summarised: 10000 (all trials)
## * No common control arm
## * Selection strategy: no selection if no superior arm
## * Treatment effect compared to: no comparison
##
## Performance metrics (using posterior estimates from final analysis [all patients]):
## * Sample sizes: mean 6710.1 (SD: 2793.2) | median 6700.0 (IQR: 4450.0 to 9950.0)
[range: 700.0 to 10000.0]
## * Total summarised outcomes: mean 1764.7 (SD: 736.5) | median 1790.0 (IQR: 1193.0 to
2560.0) [range: 163.0 to 2811.0]
## * Total summarised outcome rates: mean 0.263 (SD: 0.007) | median 0.263 (IQR: 0.259 to
0.267) [range: 0.229 to 0.307]
## * Conclusive: 77.5%
## * Superiority: 56.5%
## * Equivalence: 21.1%
## * Futility: 0.0% [not assessed]
## * Inconclusive at max sample size: 22.5%
## * Selection probabilities: Arm A: 55.9% | Arm B: 0.2% | Arm C: 0.3% | None: 43.5%
## * RMSE / MAE: 0.01504 / 0.00654
## * RMSE / MAE treatment effect: not estimated / not estimated
## * Ideal design percentage: 99.1%
##
## Simulation details:
## * Simulation time: 10.2 mins
## * Base random seed: 4137
## * Credible interval width: 95%
## * Number of posterior draws: 10000
## * Estimation method: posterior medians with MAD-SDs
##
##
## ##################################################
## Performance metrics for scenario: A 25.0 - B 22.5 - C 27.5
## Multiple simulation results: generic binomially distributed outcome trial
## * Undesirable outcome
## * Number of simulations: 10000
## * Number of simulations summarised: 10000 (all trials)
## * No common control arm
## * Selection strategy: no selection if no superior arm
```

```
## * Treatment effect compared to: no comparison
##
## Performance metrics (using posterior estimates from final analysis [all patients]):
## * Sample sizes: mean 5052.5 (SD: 2550.0) | median 4700.0 (IQR: 3200.0 to 6950.0)
[range: 700.0 to 10000.0]
## * Total summarised outcomes: mean 1215.1 (SD: 606.9) | median 1141.0 (IQR: 750.0 to
1637.0) [range: 151.0 to 2657.0]
## * Total summarised outcome rates: mean 0.242 (SD: 0.008) | median 0.241 (IQR: 0.236 to
0.246) [range: 0.211 to 0.296]
## * Conclusive: 95.2%
## * Superiority: 74.3%
## * Equivalence: 20.9%
## * Futility: 0.0% [not assessed]
## * Inconclusive at max sample size: 4.8%
## * Selection probabilities: Arm A: 0.6% | Arm B: 73.7% | Arm C: 0.0% | None: 25.7%
## * RMSE / MAE: 0.01396 / 0.00600
## * RMSE / MAE treatment effect: not estimated / not estimated
## * Ideal design percentage: 99.6%
##
## Simulation details:
## * Simulation time: 6.93 mins
## * Base random seed: 4138
## * Credible interval width: 95%
## * Number of posterior draws: 10000
## * Estimation method: posterior medians with MAD-SDs
##
##
## ##################################################
## Performance metrics for scenario: A 25.0 - B 30.0 - C 27.5
## Multiple simulation results: generic binomially distributed outcome trial
## * Undesirable outcome
## * Number of simulations: 10000
## * Number of simulations summarised: 10000 (all trials)
## * No common control arm
## * Selection strategy: no selection if no superior arm
## * Treatment effect compared to: no comparison
##
## Performance metrics (using posterior estimates from final analysis [all patients]):
## * Sample sizes: mean 5286.6 (SD: 2634.3) | median 4950.0 (IQR: 3200.0 to 7200.0)
[range: 700.0 to 10000.0]
## * Total summarised outcomes: mean 1403.0 (SD: 693.1) | median 1327.0 (IQR: 871.0 to
1906.0) [range: 167.0 to 2851.0]
## * Total summarised outcome rates: mean 0.266 (SD: 0.008) | median 0.266 (IQR: 0.261 to
0.271) [range: 0.237 to 0.326]
## * Conclusive: 93.1%
## * Superiority: 73.0%
## * Equivalence: 20.1%
## * Futility: 0.0% [not assessed]
## * Inconclusive at max sample size: 6.9%
## * Selection probabilities: Arm A: 72.3% | Arm B: 0.0% | Arm C: 0.7% | None: 27.0%
## * RMSE / MAE: 0.01509 / 0.00618
## * RMSE / MAE treatment effect: not estimated / not estimated
## * Ideal design percentage: 99.5%
```

```
##
## Simulation details:
## * Simulation time: 7.41 mins
## * Base random seed: 4139
## * Credible interval width: 95%
## * Number of posterior draws: 10000
## * Estimation method: posterior medians with MAD-SDs
##
##
## #####################################################
## Performance metrics for scenario: A 25.0 - B 20.0 - C 27.5
## Multiple simulation results: generic binomially distributed outcome trial
## * Undesirable outcome
## * Number of simulations: 10000
## * Number of simulations summarised: 10000 (all trials)
## * No common control arm
## * Selection strategy: no selection if no superior arm
## * Treatment effect compared to: no comparison
##
## Performance metrics (using posterior estimates from final analysis [all patients]):
## * Sample sizes: mean 2349.8 (SD: 1282.6) | median 2200.0 (IQR: 1450.0 to 2950.0)
[range: 700.0 to 9700.0]
## * Total summarised outcomes: mean 539.8 (SD: 286.6) | median 478.0 (IQR: 332.0 to
689.0) [range: 133.0 to 2166.0]
## * Total summarised outcome rates: mean 0.232 (SD: 0.011) | median 0.231 (IQR: 0.224 to
0.238) [range: 0.189 to 0.296]
## * Conclusive: 100.0%
## * Superiority: 99.8%
## * Equivalence: 0.2%
## * Futility: 0.0% [not assessed]
## * Inconclusive at max sample size: 0.0%
## * Selection probabilities: Arm A: 0.0% | Arm B: 99.8% | Arm C: 0.0% | None: 0.2%
## * RMSE / MAE: 0.01579 / 0.00825
## * RMSE / MAE treatment effect: not estimated / not estimated
## * Ideal design percentage: 100.0%
##
## Simulation details:
## * Simulation time: 3.29 mins
## * Base random seed: 4140
## * Credible interval width: 95%
## * Number of posterior draws: 10000
## * Estimation method: posterior medians with MAD-SDs
##
##
## #####################################################
## Performance metrics for scenario: A 25.0 - B 22.5 - C 22.5
## Multiple simulation results: generic binomially distributed outcome trial
## * Undesirable outcome
## * Number of simulations: 10000
## * Number of simulations summarised: 10000 (all trials)
## * No common control arm
## * Selection strategy: no selection if no superior arm
## * Treatment effect compared to: no comparison
```

```
##
## Performance metrics (using posterior estimates from final analysis [all patients]):
## * Sample sizes: mean 6239.5 (SD: 2386.3) | median 5700.0 (IQR: 4450.0 to 8200.0)
[range: 700.0 to 10000.0]
## * Total summarised outcomes: mean 1434.7 (SD: 553.9) | median 1329.0 (IQR: 1001.0 to
1900.0) [range: 139.0 to 2429.0]
## * Total summarised outcome rates: mean 0.230 (SD: 0.006) | median 0.230 (IQR: 0.226 to
0.234) [range: 0.198 to 0.274]
## * Conclusive: 87.2%
## * Superiority: 13.7%
## * Equivalence: 73.6%
## * Futility: 0.0% [not assessed]
## * Inconclusive at max sample size: 12.8%
## * Selection probabilities: Arm A: 0.1% | Arm B: 6.6% | Arm C: 7.0% | None: 86.3%
## * RMSE / MAE: 0.02270 / 0.01309
## * RMSE / MAE treatment effect: not estimated / not estimated
## * Ideal design percentage: 99.5%
##
## Simulation details:
## * Simulation time: 9.35 mins
## * Base random seed: 4141
## * Credible interval width: 95%
## * Number of posterior draws: 10000
## * Estimation method: posterior medians with MAD-SDs
##
##
## ##################################################
## Performance metrics for scenario: A 25.0 - B 30.0 - C 22.5
## Multiple simulation results: generic binomially distributed outcome trial
## * Undesirable outcome
## * Number of simulations: 10000
## * Number of simulations summarised: 10000 (all trials)
## * No common control arm
## * Selection strategy: no selection if no superior arm
## * Treatment effect compared to: no comparison
##
## Performance metrics (using posterior estimates from final analysis [all patients]):
## * Sample sizes: mean 4715.6 (SD: 2525.0) | median 4450.0 (IQR: 2700.0 to 6450.0)
[range: 700.0 to 10000.0]
## * Total summarised outcomes: mean 1135.3 (SD: 595.9) | median 1062.0 (IQR: 666.0 to
1519.0) [range: 149.0 to 2523.0]
## * Total summarised outcome rates: mean 0.243 (SD: 0.010) | median 0.242 (IQR: 0.237 to
0.247) [range: 0.211 to 0.301]
## * Conclusive: 96.4%
## * Superiority: 74.6%
## * Equivalence: 21.8%
## * Futility: 0.0% [not assessed]
## * Inconclusive at max sample size: 3.6%
## * Selection probabilities: Arm A: 0.7% | Arm B: 0.0% | Arm C: 74.0% | None: 25.4%
## * RMSE / MAE: 0.01436 / 0.00623
## * RMSE / MAE treatment effect: not estimated / not estimated
## * Ideal design percentage: 99.7%
##
```

```
## Simulation details:
## * Simulation time: 6.16 mins
## * Base random seed: 4142
## * Credible interval width: 95%
## * Number of posterior draws: 10000
## * Estimation method: posterior medians with MAD-SDs
##
##
## #####################################################
## Performance metrics for scenario: A 25.0 - B 20.0 - C 22.5
## Multiple simulation results: generic binomially distributed outcome trial
## * Undesirable outcome
## * Number of simulations: 10000
## * Number of simulations summarised: 10000 (all trials)
## * No common control arm
## * Selection strategy: no selection if no superior arm
## * Treatment effect compared to: no comparison
##
## Performance metrics (using posterior estimates from final analysis [all patients]):
## * Sample sizes: mean 4788.2 (SD: 2427.8) | median 4450.0 (IQR: 2950.0 to 6450.0)
[range: 700.0 to 10000.0]
## * Total summarised outcomes: mean 1032.2 (SD: 517.0) | median 965.0 (IQR: 641.0 to
1373.2) [range: 134.0 to 2362.0]
## * Total summarised outcome rates: mean 0.217 (SD: 0.008) | median 0.216 (IQR: 0.211 to
0.221) [range: 0.182 to 0.261]
## * Conclusive: 96.7%
## * Superiority: 75.5%
## * Equivalence: 21.2%
## * Futility: 0.0% [not assessed]
## * Inconclusive at max sample size: 3.3%
## * Selection probabilities: Arm A: 0.0% | Arm B: 74.9% | Arm C: 0.6% | None: 24.5%
## * RMSE / MAE: 0.01322 / 0.00595
## * RMSE / MAE treatment effect: not estimated / not estimated
## * Ideal design percentage: 99.6%
##
## Simulation details:
## * Simulation time: 6.64 mins
## * Base random seed: 4143
## * Credible interval width: 95%
## * Number of posterior draws: 10000
## * Estimation method: posterior medians with MAD-SDs
##
##
## #####################################################
## Performance metrics for scenario: A 25.0 - B 30.0 - C 30.0
## Multiple simulation results: generic binomially distributed outcome trial
## * Undesirable outcome
## * Number of simulations: 10000
## * Number of simulations summarised: 10000 (all trials)
## * No common control arm
## * Selection strategy: no selection if no superior arm
## * Treatment effect compared to: no comparison
##
```

```
## Performance metrics (using posterior estimates from final analysis [all patients]):
## * Sample sizes: mean 3204.8 (SD: 1754.4) | median 2950.0 (IQR: 1950.0 to 4200.0)
[range: 700.0 to 10000.0]
## * Total summarised outcomes: mean 881.9 (SD: 480.4) | median 795.0 (IQR: 518.8 to
1155.0) [range: 162.0 to 3062.0]
## * Total summarised outcome rates: mean 0.276 (SD: 0.010) | median 0.275 (IQR: 0.270 to
0.282) [range: 0.227 to 0.341]
## * Conclusive: 99.8%
## * Superiority: 99.3%
## * Equivalence: 0.5%
## * Futility: 0.0% [not assessed]
## * Inconclusive at max sample size: 0.2%
## * Selection probabilities: Arm A: 99.2% | Arm B: 0.0% | Arm C: 0.0% | None: 0.7%
## * RMSE / MAE: 0.01544 / 0.00771
## * RMSE / MAE treatment effect: not estimated / not estimated
## * Ideal design percentage: 99.9%
##
## Simulation details:
## * Simulation time: 4.59 mins
## * Base random seed: 4144
## * Credible interval width: 95%
## * Number of posterior draws: 10000
## * Estimation method: posterior medians with MAD-SDs
##
##
## ##################################################
## Performance metrics for scenario: A 25.0 - B 20.0 - C 30.0
## Multiple simulation results: generic binomially distributed outcome trial
## * Undesirable outcome
## * Number of simulations: 10000
## * Number of simulations summarised: 10000 (all trials)
## * No common control arm
## * Selection strategy: no selection if no superior arm
## * Treatment effect compared to: no comparison
##
## Performance metrics (using posterior estimates from final analysis [all patients]):
## * Sample sizes: mean 2211.2 (SD: 1263.4) | median 1950.0 (IQR: 1200.0 to 2950.0)
[range: 700.0 to 10000.0]
## * Total summarised outcomes: mean 512.3 (SD: 279.7) | median 449.0 (IQR: 299.0 to
651.0) [range: 139.0 to 2168.0]
## * Total summarised outcome rates: mean 0.235 (SD: 0.013) | median 0.234 (IQR: 0.226 to
0.243) [range: 0.193 to 0.307]
## * Conclusive: 100.0%
## * Superiority: 99.8%
## * Equivalence: 0.2%
## * Futility: 0.0% [not assessed]
## * Inconclusive at max sample size: 0.0%
## * Selection probabilities: Arm A: 0.1% | Arm B: 99.7% | Arm C: 0.0% | None: 0.2%
## * RMSE / MAE: 0.01617 / 0.00868
## * RMSE / MAE treatment effect: not estimated / not estimated
## * Ideal design percentage: 100.0%
##
## Simulation details:
```

```
## * Simulation time: 2.99 mins
## * Base random seed: 4145
## * Credible interval width: 95%
## * Number of posterior draws: 10000
## * Estimation method: posterior medians with MAD-SDs
##
##
## ##################################################
## Performance metrics for scenario: A 25.0 - B 20.0 - C 20.0
## Multiple simulation results: generic binomially distributed outcome trial
## * Undesirable outcome
## * Number of simulations: 10000
## * Number of simulations summarised: 10000 (all trials)
## * No common control arm
## * Selection strategy: no selection if no superior arm
## * Treatment effect compared to: no comparison
##
## Performance metrics (using posterior estimates from final analysis [all patients]):
## * Sample sizes: mean 4649.7 (SD: 1730.2) | median 4200.0 (IQR: 3700.0 to 5450.0)
[range: 700.0 to 10000.0]
## * Total summarised outcomes: mean 954.9 (SD: 352.6) | median 883.0 (IQR: 755.0 to
1118.0) [range: 125.0 to 2171.0]
## * Total summarised outcome rates: mean 0.206 (SD: 0.008) | median 0.205 (IQR: 0.201 to
0.210) [range: 0.179 to 0.257]
## * Conclusive: 99.0%
## * Superiority: 13.8%
## * Equivalence: 85.1%
## * Futility: 0.0% [not assessed]
## * Inconclusive at max sample size: 1.0%
## * Selection probabilities: Arm A: 0.0% | Arm B: 6.8% | Arm C: 7.0% | None: 86.2%
## * RMSE / MAE: 0.02397 / 0.01508
## * RMSE / MAE treatment effect: not estimated / not estimated
## * Ideal design percentage: 99.9%
##
## Simulation details:
## * Simulation time: 6.12 mins
## * Base random seed: 4146
## * Credible interval width: 95%
## * Number of posterior draws: 10000
## * Estimation method: posterior medians with MAD-SDs

# Print and save key results
key_results

##        A     B     C size pr_concl pr_sup pr_equi pr_err_sup
## 1  25.0% 25.0% 25.0% 7932    66.4%   4.8%   61.6%       4.8%
## 2  25.0% 27.5% 25.0% 6496    85.2%  14.6%   70.6%      14.6%
## 3  25.0% 22.5% 25.0% 6473    81.5%  59.7%   21.8%       0.4%
## 4  25.0% 30.0% 25.0% 5304    97.1%  14.1%   83.0%      14.1%
## 5  25.0% 20.0% 25.0% 2871   100.0%  99.6%    0.4%       0.0%
## 6  25.0% 27.5% 27.5% 6710    77.5%  56.5%   21.1%       0.5%
## 7  25.0% 22.5% 27.5% 5052    95.2%  74.3%   20.9%       0.6%
## 8  25.0% 30.0% 27.5% 5287    93.1%  73.0%   20.1%       0.7%
```

```
## 9  25.0% 20.0% 27.5% 2350   100.0%  99.8%    0.2%       0.0%
## 10 25.0% 22.5% 22.5% 6239    87.2%  13.7%   73.6%      13.7%
## 11 25.0% 30.0% 22.5% 4716    96.4%  74.6%   21.8%       0.7%
## 12 25.0% 20.0% 22.5% 4788    96.7%  75.5%   21.2%       0.6%
## 13 25.0% 30.0% 30.0% 3205    99.8%  99.3%    0.5%       0.1%
## 14 25.0% 20.0% 30.0% 2211   100.0%  99.8%    0.2%       0.1%
## 15 25.0% 20.0% 20.0% 4650    99.0%  13.8%   85.1%      13.8%

write.csv2(
  key_results,
  file = paste0(dir_out, "Performance primary calibrated 15 scenarios.csv"),
  row.names = FALSE
)
```

Log date and session info

Save date and R/package versions for reproducibility:

```
date()

## [1] "Mon Aug 25 10:49:02 2025"

sessionInfo()

## R version 4.4.1 (2024-06-14 ucrt)
## Platform: x86_64-w64-mingw32/x64
## Running under: Windows 11 x64 (build 22631)
##
## Matrix products: default
##
##
## locale:
## [1] LC_COLLATE=Danish_Denmark.utf8  LC_CTYPE=Danish_Denmark.utf8
## [3] LC_MONETARY=Danish_Denmark.utf8 LC_NUMERIC=C
## [5] LC_TIME=Danish_Denmark.utf8
##
## time zone: Europe/Copenhagen
## tzcode source: internal
##
## attached base packages:
## [1] stats     graphics  grDevices utils     datasets  methods   base
##
## other attached packages:
## [1] adaptr_1.4.0
##
## loaded via a namespace (and not attached):
##  [1] compiler_4.4.1    fastmap_1.2.0     cli_3.6.5         parallel_4.4.1
##  [5] tools_4.4.1       htmltools_0.5.8.1 rstudioapi_0.17.1 yaml_2.3.10
##  [9] rmarkdown_2.29    knitr_1.50        xfun_0.52         digest_0.6.37
## [13] rlang_1.1.6       evaluate_1.0.4
```
