# Supplementary material for: Designing and Evaluating Bayesian Advanced Adaptive Randomised Clinical Trials: A Practical Guide
Source: Pharm Stat. 2025 Oct 9;24(6):e70042. doi: 10.1002/pst.70042 (PMC12509790; doi:10.1002/pst.70042)
Supplement: Supplementary file 3 — Appendix C. Descriptions of additional examples and the corresponding code are included in Appendices C and D (Appendix C is a formatted PDF including code, explanation, and outputs/figures). [file PST-24-0-s003.pdf]

Designing and evaluating Bayesian advanced adaptive randomised clinical trials: a practical guide

Appendix C

Anders Granholm[1,2], Aksel Karl Georg Jensen[1,2], Theis Lange[2],
Anders Perner[1,3], Morten Hylander Møller[1,3], and Benjamin Skov Kaas-Hansen[1,2]

[1] Department of Intensive Care 4131, Copenhagen University Hospital – Rigshospitalet, Copenhagen, Denmark
[2] Section of Biostatistics, Department of Public Health, University of Copenhagen, Copenhagen, Denmark
[3] Department of Clinical Medicine, Faculty of Health and Medical Sciences, University of Copenhagen, Copenhagen, Denmark

Correspondence: Anders Granholm (anders.granholm@regionh.dk)

Appendix C

This supplementary appendix includes supplementary examples of various trial designs along with explanations. **Appendix D** essentially contains the same code, but as a raw R script with explanation in comments and without outputs.

For additional details and examples, see the primary manuscript and the ***adaptr*** package documentation available at: https://inceptdk.github.io/adaptr/.

Setup

```
# Load adaptr
library(adaptr)

## Loading 'adaptr' package v1.4.0.
## For instructions, type 'help("adaptr")'
## or see https://inceptdk.github.io/adaptr/.

# Load ggplot2 (used for illustrations in this supplement)
library(ggplot2)
```

Example 1: Design using a common control arm

In this example, we specify a trial design specification with four arms with one arm (`'Standard'`) used as a common control arm to which the other arms are compared pairwise. If one of the other arms is superior to the initial common control in an adaptive analysis, the initial common control arm will be dropped and the arm deemed superior to this arm will become the new common control.

The design uses the special argument `control_prob_fixed`, which is set to `'sqrt-based'`. This means that the trial will use a fixed allocation probability to the common control arm, which is defined as the square root to the number of currently active non-control arms to 1 (for each active non-control arm). This will similarly be used for 'new' common control arms.

All non-control arms use response-adaptive randomisation, limited at minimum 15% (re-scaled proportionately when arms are dropped; not specified for the control arm, as this uses a fixed allocation probability) and with a *softening factor* of 0.5 applied.

The trial design specifies stopping rules for inferiority/superiority, but these could be calibrated if desired (as described in the main text). The trial design allows dropping non-control arms for practical equivalence if the probability that the absolute difference between a non-control arm and the current common control is less than 2.5%-points exceeds 90%. Similarly, non-control arms will be dropped for futility if the probability that they are NOT superior by at least 2.5%-points exceeds 90%. Practical equivalence and futility will only be assessed in comparisons to the initial common control.

In the scenario here, outcome distributions are identical in all arms (i.e., this represents a *null* scenario). The `setup_trial_binom()` function is used.

```
design_common_control_null_scenario <- setup_trial_binom(
  # Arms and scenario
  arms = c("Standard", "Intervention A", "Intervention B", "Intervention C"),
  control = "Standard", # Common control arm
  true_ys = rep(0.25, 4),
  highest_is_best = FALSE,
  # Allocation rules
  min_probs = c(NA, 0.15, 0.15, 0.15),
  control_prob_fixed = "sqrt-based",
  rescale_probs = "limits",
  soften_power = 0.5,
  # participants with data/randomised at each analysis
  data_looks = seq(from = 500, to = 10000, by = 250),
  randomised_at_looks = pmin(seq(from = 500, to = 10000, by = 250) + 200, 10000),
  # stopping rules
  inferiority = 0.01, # default
  superiority = 0.99, # default
  equivalence_prob = 0.9,
  equivalence_diff = 0.025,
  equivalence_only_first = TRUE, # Only assessed against initial common control
  futility_prob = 0.9,
  futility_diff = 0.025,
  futility_only_first = TRUE, # Only assessed against initial common control
  # Posterior draws
  n_draws = 10000
)

# Print
design_common_control_null_scenario

## Trial specification: generic binomially distributed outcome trial
## * Undesirable outcome
## * Common control arm: Standard
```

```
## * Control arm probability fixed at 0.366 (for 4 arms), 0.414 (for 3 arms), 0.5 (for 2 arms)
## * Best arms: Standard and Intervention A and Intervention B and Intervention C
##
## Arms, true outcomes, starting allocation probabilities
## and allocation probability limits (min/max_probs rescaled):
##            arms true_ys start_probs fixed_probs min_probs max_probs
##        Standard    0.25       0.366       0.366        NA        NA
##  Intervention A    0.25       0.211          NA      0.15        NA
##  Intervention B    0.25       0.211          NA      0.15        NA
##  Intervention C    0.25       0.211          NA      0.15        NA
##
## Maximum sample size: 10000
## Maximum number of data looks: 39
## Planned data looks after:  500, 750, 1000, 1250, 1500, 1750, 2000, 2250, 2500, 2750, 3000, 3250, 3500, 3750, 4000, 4250, 4500, 4750, 5000, 5250, 5500, 5750, 6000, 6250, 6500, 6750, 7000, 7250, 7500, 7750, 8000, 8250, 8500, 8750, 9000, 9250, 9500, 9750, 10000 patients have reached follow-up
## Number of patients randomised at each look:  700, 950, 1200, 1450, 1700, 1950, 2200, 2450, 2700, 2950, 3200, 3450, 3700, 3950, 4200, 4450, 4700, 4950, 5200, 5450, 5700, 5950, 6200, 6450, 6700, 6950, 7200, 7450, 7700, 7950, 8200, 8450, 8700, 8950, 9200, 9450, 9700, 9950, 10000
##
## Superiority threshold: 0.99 (all analyses)
## Inferiority threshold: 0.01 (all analyses)
## Equivalence threshold: 0.9 (all analyses) (only checked for first control)
## Absolute equivalence difference: 0.025
## Futility threshold: 0.9 (all analyses) (only checked for first control)
## Absolute futility difference (in beneficial direction): 0.025
## Soften power for all analyses: 0.5
```

Example 2: Design using custom complex outcome-generating function

Example introduction

This example specifies a trial design using the primary `setup_trial()` function, including custom outcome-generation and analysis functions, and explicit considerations regarding outcome data-lag and expected inclusion rates.

This example is inspired by an upcoming domain on the upcoming adaptive platform trial **INCEPT** (*The Intensive Care Platform Trial*, see INCEPT.dk). This will be a closed domain (i.e., arm adding is not permitted), and will thus to a large extent behave similar to a stand-alone advanced adaptive trial (see DOI: 10.1038/s41573-019-0034-3 for additional explanation).

As the outcome distribution considered is complex, the example is likewise relatively complex, to ensure that the outcome generation function provides a realistic outcome distribution.

The example uses two intervention arms, and the outcome considered here is ***days alive without life support at day 30***: a beneficial count outcome with a range of 0-29 (as participants will be on life support at inclusion). This outcome typically has substantial zero-inflation, which is considered when the outcome generation function is specified. Additional details on this and similar outcomes can be found elsewhere (DOI: 10.1186/s12874-023-01963-z).

Distribution description and specification

Here, data are generated from a 'hurdle-beta' two-part distribution (often referred to as a zero-inflated beta distribution, which is technically less correct) consisting of two sub-distributions. First, a binomial distribution controls the proportion of 0'es, which is set to 35% in this example. Second, a beta distribution controls the number of days in those with >0 days. As beta distributions model proportions (without including 0'es or 1's, i.e., only values >0% and <100% can be generated using this distribution), and as we only want 0'es to be generated by the binomial sub-distribution, the proportions obtained from the beta distribution are multiplied by the maximum possible number of days (29) and rounded upwards to the nearest whole number. This ensures that the beta sub-distribution does not generate any 0'es, but is able to generate the maximum value (29). While this will increase the overall mean of the outcome distribution generated slightly compared to the intended overall mean, this is unlikely to have any substantial influence on how the generated distribution resembles the intended reference distribution, and is considered smaller than the uncertainty associated with defining how a complex distribution like this is expected to be in a new trial population.

We want the overall distribution to have a mean number of days of 14; meaning that the mean number of days in those with >0 days should be approximately `14 / (1 - 0.35) = 21.5`, corresponding to a proportion of days of approximately 74.3% in those with >0 days. The variance component of the beta distribution here is set to be 0.05, which is roughly similar to the variance used in the upcoming INCEPT domain that this example is based upon (this was derived by fitting a beta distribution to the distribution of this outcome in those with >0 days in a previous trial that was considered likely to have a similar outcome distribution as expected here).

Of note, we have defined the Beta distribution using the *mean and variance* parameterisation as this is the easiest to use and interpret in this context. The functions built into R use the *alpha and beta* parametrisation (called *shape 1 and shape2*, respectively, in R), and thus we convert the parameters (see, e.g., the Beta distribution article on Wikipedia for additional explanation, including on the parametrisations and formulae for converting from one parametrisation to another):

Define Beta distribution alpha and beta parameters:

```
beta_mean <- 0.743 # Mean in those with >0 days in proportion scale
beta_var <- 0.05

beta_alpha <- abs((beta_mean * (beta_var + beta_mean^2 - beta_mean)) / beta_var)
beta_beta <- abs(( (beta_var + beta_mean^2 - beta_mean) * (beta_mean - 1) ) / beta_var)

cat("Beta distribution paramaters:",
    "\n- alpha (shape1):", beta_alpha,
    "\n- beta (shape2):", beta_beta)

## Beta distribution paramaters:
## - alpha (shape1): 2.094532
## - beta (shape2): 0.7244881
```

For illustrative purposes, we sample 100,000 values from this distribution using the values described above and similar rounding. As seen, the overall mean is slightly larger than the 14 days specified above due to rounding; this is considered acceptable (and the resulting distribution used below).

```
set.seed(4131) # Reproducibility
# Sample each participants' chance of 0 days
sample_0_days <- rbinom(n = 100000, size = 1, prob = 0.35)
# Sample each participants' number of days if >0 days
sample_1p_days <- rbeta(
  n = 100000,
  shape1 = beta_alpha,
  shape2 = beta_beta,
)
# Combine both distributions and multiply and round to number of days
sample_days <- ceiling((1 - sample_0_days) * sample_1p_days * 29)

# Plot
ggplot(data = data.frame(days = sample_days), aes(x = days)) +
  geom_histogram(bins = 30) +
  scale_x_continuous(name = "Days alive without life support at day 30",
                     breaks = 0:5 * 5, expand = c(0, 0)) +
  scale_y_continuous(name = NULL, labels = function(x) (scales::percent(x / 100000)),
                     limits = c(0, 0.4 * 100000), expand = c(0, 0)) +
  labs(subtitle = paste("Mean:", round(mean(sample_days), 1),
                        "- Median:", round(median(sample_days), 1),
                        "- Interquartile range:", round(quantile(sample_days, probs =
0.25), 1),
                        "to", round(quantile(sample_days, probs = 0.75), 1), "days")) +
  theme_bw()
```

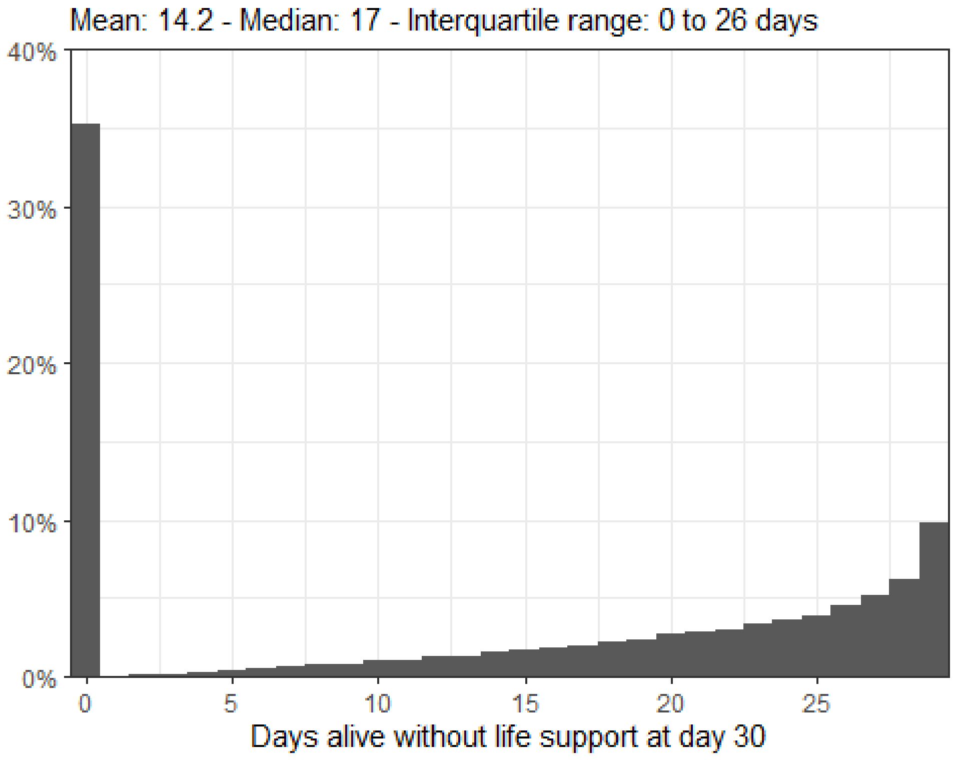

Defining outcome-generating function

Now, we specify the function to generate outcomes in the scenario with no differences between the two arms (denoted **'Arm A'** and **'Arm B'**), but in a format that makes this easy to change for scenarios with differences present. Instead of having one custom function for each scenario, advanced users may want to make a 'function factory' (https://adv-r.hadley.nz/function-factories.html), but this is not done here to avoid overly complicating the example.

The requirements for outcome generation functions in `adaptr` are described in detail in the `setup_trial()` function documentation. Although outcome distributions are identical in both arms in this scenario, we setup the function so that it loops through each arm, making it easy to change the most important distributional parameters, i.e., the proportion of zeroes and the proportion of days in those with > 0 days. Here, we choose to hardcode the beta distribution variance, as it is difficult to specify how this is expected to change between clinical scenarios. We thus generally consider it reasonable to assume that the variance parameter is unchanged between clinical scenarios, and that differences are mediated through changes to the proportions of zero days and/or the mean number of days for those with >0 days.

```
# The only argument required is 'allocs', a character vector with all newly
# allocated participants that outcomes should be generated for
# The only return value should be a vector with outcomes (encoded as numerical
# values) for each participant in the same order as allocs
get_ys_hurdlebeta_days29_no_difference <- function(allocs) {
  # Setup empty return vector with outcomes
  y <- numeric(length(allocs))
  # Named vector of proportion of zeroes in each arm
  prop0 <- c("Arm A" = 0.35, "Arm B" = 0.35)
  # Named vector of the proportion of days in each arm in those with >0 days
  prop_days_1p <- c("Arm A" = 0.743, "Arm B" = 0.743)
  # Generate outcomes for each arm
  for (arm in c("Arm A", "Arm B")) {
    ii <- which(allocs == arm) # Indices of participants in current arm
    y[ii] <- ceiling(
      (1 - rbinom(n = length(ii), size = 1, prob = prop0[arm])) * # Zeroes
        rbeta(n = length(ii),
              shape1 = abs((prop_days_1p[arm] * (0.05 + prop_days_1p[arm]^2 -
prop_days_1p[arm])) / 0.05),
              shape2 = abs(( (0.05 + prop_days_1p[arm]^2 - prop_days_1p[arm]) *
(prop_days_1p[arm] - 1) ) / 0.05)
        ) * 29) # Multiply and round
  }
  # Return outcomes
  y
}
```

Randomly sampling from the distribution using this function (with participants in both arms), generates similar distributions as the one illustrated above:

```
set.seed(2024) # Reproducibility
ggplot(data = data.frame(
  days = get_ys_hurdlebeta_days29_no_difference(allocs = rep(c("Arm A", "Arm B"), 5000))),
  aes(x = days)) +
  geom_histogram(bins = 30) +
```

```
  scale_x_continuous(name = "Days alive without life support at day 30",
                     breaks = 0:5 * 5, expand = c(0, 0)) +
  scale_y_continuous(name = NULL, labels = function(x) (scales::percent(x / 10000)),
                     limits = c(0, 0.4 * 10000), expand = c(0, 0)) +
  labs(subtitle = paste("Mean:", round(mean(sample_days), 1),
                        "- Median:", round(median(sample_days), 1),
                        "- Interquartile range:", round(quantile(sample_days, probs =
0.25), 1),
                        "to", round(quantile(sample_days, probs = 0.75), 1), "days")) +
  theme_bw()
```

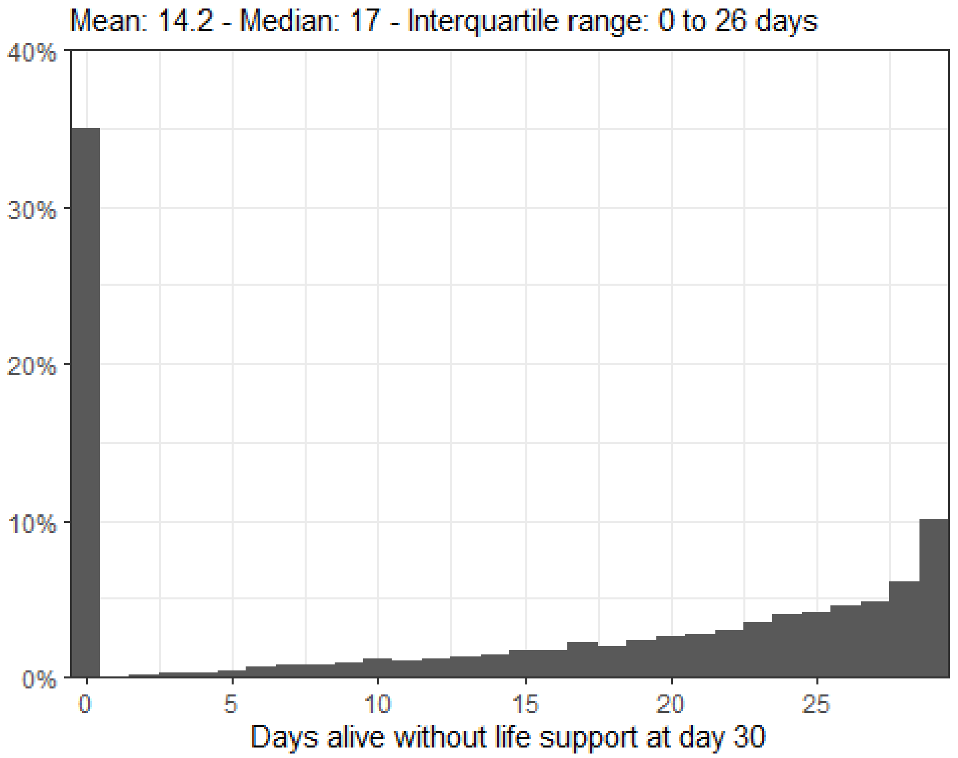

Of note, when using an outcome with a complex distribution like this and specifying scenarios with differences present, these may be specified to either only affect one part of the distribution (e.g., the proportion of 0'es or the means in those with values >0 in this example) or multiple parts, and, if so, to which extent differences are mediated by changes in the different parts of the distribution. It may be relevant to assess multiple sets of scenarios assessing differences mediated on different parts or combinations of parts of the distribution, ideally with one set of scenarios being the primary. Similarly, when doing sensitivity analyses of trial designs challenging the assumed outcome distributions, it may be relevant to assess the influence of similar overall changes in either direction but mediated via different parts or combinations of parts of the overall distribution.

Defining analysis function

In addition to the custom outcome-generating function, a custom function used to conduct the analysis and return the posterior draws is required when using the `setup_trial()` function. Here, we specify

such a function in the correct format (additional details on the function arguments and required output is provided in the `setup_trial()` documentation).

Days alive without life support and similar outcomes may be analysed using different approaches, as discussed elsewhere (DOI: 10.1186/s12874-023-01963-z).

In this example, we are interested in the overall means in each arm. In a 'real' trial, one option is to analyse this using linear regression, as modelling the entire distribution is of less interest than 'just' estimating the means in each arm and their differences. For simplicity, we thus simply generate posterior draws from normal distributions with means corresponding to the overall mean in each arm, and standard deviations defined as the standard errors of the mean, using the conventional approach to calculation, i.e., the standard deviation in each arm divided by the square root to the number of participants in each arm minus 1. While this is an approximation that requires moderately large samples to provide adequately accurate measures of the standard errors of the means and thus the posterior distributions, we consider it adequate in this example as the first adaptive analysis is conducted after 500 participants have follow-up data available. In this example, we use no prior information in each arm, i.e., technically we use improper and completely flat priors. The function defined below corresponds to the simplified analysis function internally used by `setup_trial_norm()` in the current version of `adaptr`, just with added explanation:

```
# The arguments that must be as specified here:
# - arms: character vector of all unique and currently active arms
# - allocs: character vector with allocations of all participants (in order of
randomisation)
# - ys: character vector with outcomes of each participant (same order as allocs)
# - control: single character string, common control arm or NULL if none
# - n_draws: single integer, number of posterior draws in each arm
# Of note, even if no common control arm is used, the 'control' argument must be
# included as the other functions in the package supply this argument to all
# outcome-generating functions, as it may sometimes be relevant to use.
# The function must return a matrix of posterior draws (as numerical values)
# with one named column per currently active arm and with the number of rows
# corresponding to n_draws.
get_draws_norm_noprior <- function(arms, allocs, ys, control, n_draws) {
  draws <- list() # Prepare list to store results in
  for (arm in arms) { # Loop through all arms
    ii <- which(allocs == arm) # Indices of participants randomised to current arm
    n <- length(ii)
    if (n > 1){ # Return draws using the method described above if enough participants
randomised
      draws[[arm]] <- rnorm(n_draws, mean = mean(ys[ii]), sd = sd(ys[ii]) / sqrt(n - 1))
    } else {
      # Too few patients randomised - return extreme uncertainty based on the data
      # This is necessary to avoid errors if too few patients have been randomised to this
arm yet
      draws[[arm]] <- rnorm(n_draws, mean = mean(ys), sd = 1000 * (max(ys) - min(ys)))
    }
  }
  do.call(cbind, draws) # Bind each vector contained in the list to a matrix
}
```

Trial design specification

We can now setup the complete trial specification using the functions defined above (these could also be directly specified within the `setup_trial()` call). Importantly, the `setup_trial()` function validates that the two custom functions run when supplied with the correct arguments and return outputs in the correct format, but it is the responsibility of the user to ensure that they otherwise work as intended (i.e., that the internal calculations are correct).

```
design_complex_outcome_no_difference <- setup_trial(
  # Arm settings
  arms = c("Arm A", "Arm B"),
  control = NULL, # No common control
  # True overall means in each arm, using the means estimated from the combined
  # distribution after rounding (slightly higher than 14 days)
  true_ys = c(14.2, 14.2),
  # Custom functions to generate outcomes and posterior draws
  fun_y_gen = get_ys_hurdlebeta_days29_no_difference,
  fun_draws = get_draws_norm_noprior,
  highest_is_best = TRUE, # More days are better
  # Function to estimate the raw estimates (i.e., not from the posteriors) if
  # desired when calculating certain performance metrics - the default is to use
  # the posterior estimates in calculations, but this must always be specified
  fun_raw_est = mean, # Use the raw means
  # Use posterior medians (of the distribution of posterior means)for calculating
performance metrics
  robust = TRUE,
  # Allocation rules
  start_probs = c(0.5, 0.5),, # Initial equal allocation
  fixed_probs = c(0.5, 0.5), # Fixed equal allocation
  # Participants with data available/randomised at each analysis
  data_looks = seq(from = 500, to = 10000, by = 250),
  randomised_at_looks = c(seq(from = 700, to = 9950, by = 250), 10000),
  # Stopping rules
  inferiority = 0.01,
  superiority = 0.99,
  equivalence_prob = ifelse(seq(from = 500, to = 10000, by = 250) < 1500, 1, 0.9),
  equivalence_diff = 0.025,
)

# Print
design_complex_outcome_no_difference

## Trial specification
## * Desirable outcome
## * No common control arm
## * Best arms: Arm A and Arm B
##
## Arms, true outcomes, starting allocation probabilities
## and allocation probability limits:
##   arms true_ys start_probs fixed_probs min_probs max_probs
##  Arm A    14.2         0.5         0.5        NA        NA
##  Arm B    14.2         0.5         0.5        NA        NA
##
```

```
## Maximum sample size: 10000
## Maximum number of data looks: 39
## Planned data looks after:  500, 750, 1000, 1250, 1500, 1750, 2000, 2250, 2500, 2750,
3000, 3250, 3500, 3750, 4000, 4250, 4500, 4750, 5000, 5250, 5500, 5750, 6000, 6250, 6500,
6750, 7000, 7250, 7500, 7750, 8000, 8250, 8500, 8750, 9000, 9250, 9500, 9750, 10000
patients have reached follow-up
## Number of patients randomised at each look:  700, 950, 1200, 1450, 1700, 1950, 2200,
2450, 2700, 2950, 3200, 3450, 3700, 3950, 4200, 4450, 4700, 4950, 5200, 5450, 5700, 5950,
6200, 6450, 6700, 6950, 7200, 7450, 7700, 7950, 8200, 8450, 8700, 8950, 9200, 9450, 9700,
9950, 10000
##
## Superiority threshold: 0.99 (all analyses)
## Inferiority threshold: 0.01 (all analyses)
## Equivalence thresholds:
## 1, 1, 1, 1, 0.9, 0.9, 0.9, 0.9, 0.9, 0.9, 0.9, 0.9, 0.9, 0.9, 0.9, 0.9, 0.9, 0.9, 0.9,
0.9, 0.9, 0.9, 0.9, 0.9, 0.9, 0.9, 0.9, 0.9, 0.9, 0.9, 0.9, 0.9, 0.9, 0.9, 0.9, 0.9, 0.9,
0.9, 0.9
## (no common control)
## Absolute equivalence difference: 0.025
## No futility threshold (not relevant - no common control)
## Soften power for all analyses: 1 (no softening - all arms fixed)
```

Example 3: Design using custom analysis function with custom priors

Example introduction

Here, we specify a trial design using a custom function for generating posterior draws using informative priors. The example is inspired by the Empirical Meropenem versus Piperacillin/Tazobactam for Adult Patients with Sepsis (EMPRESS) trial (DOI: 10.1111/aas.14441) and a previous simulation study using `adaptr` (DOI: 10.1002/pst.2387; code included in the supplement).

The example trial uses two arms and an undesirable binary outcome, (e.g., mortality). A custom outcome-generating function is specified (as this is required by `setup_trial()`), but this function is largely similar to the function used by `setup_trial_binom()`.

Model and prior

In practice, a trial like this could be analysed using a logistic regression model with a prior on the intervention effect (i.e., the difference between arms) specified on the log odds ratio scale. Here, we want to use an informative, neutral (centred on no difference) prior conveying scepticism towards large intervention effects. In the actual analysis, such a prior could be specified as a normal distribution with mean 0 and a standard deviation of, e.g., 0.5, corresponding to a distribution on the odds ratio scale centred on 1.00 and with 95% central probability mass between 0.38 and 2.66.

The amount of information this prior conveys can be 'translated' to the number of participants that would provide the same information in a trial with equal randomisation to both arms and a specific event rate in both groups. This calculation can be done according to a specific formula (see *Greenland S, Rothman KJ. Chapter 14: introduction to categorical statistics. In: KJ Rothman, TL Lash, S Greenland, eds. Modern Epidemiology. 3rd ed. Lippincott Williams & Wilkins; 2012: 237-257.* and DOI: 10.1002/pst.2387):

```
n = 1 / (prior_SD^2) * (4 / r + 4 / (1 - r) )
```

Where `n` is the total sample size (both arms combined), `prior_SD` is the standard deviation of the desired prior (normally distributed and with mean 0), and `r` is the event probability in both arms. Thus, a prior with a standard deviation of 0.5 corresponds to the same information as approximately 85 participants in a 'previous' trial with an event rate of 25% in both arms.

For simulation purposes, we use separate, conjugate beta-binomial models for each arm (see *Lambert B. Chapter 9: conjugate priors. In: B Lambert, ed. A Student's Guide to Bayesian Statistics. 1st ed. SAGE Publications Ltd.; 2018: 237-257.*). For these models, the prior can be specified as the number of participants with the outcome and without the outcome, respectively, in each arm. We can thus use the formula specified above to 'convert' the prior that will be used for the actual analyses (specified as a normally distributed prior for the difference between arms on the log odds ratio scale) to appropriate beta priors. First, we can derive the total sample size in a trial using the combined event rate estimate across both arms at any time and the formula above. Second, we then use half of the total estimated sample size for the beta prior in each arm, which then corresponds to this number of participants in total, with the event rate and non-event rate matching the combined current event/non-event rates in the current simulation. By doing so, we ensure that the priors have equal effect on the estimates in both arms, corresponding to the 'intended' prior in the actual planned analyses of the trial being neutral.

Defining analysis function

Here, we specify the function used to generate posterior draws in both arms according to the desired prior information and the details specified above. The required format, arguments, and outputs for functions generating posterior draws have been described in the previous example.

```
fun_draws_prior <- function(arms, allocs, ys, control, n_draws) {
  # Total event rates and number of participants in a corresponding trial
  # with two arms, equal inclusion rates, and equal event rates
  prior_sd <- 0.5 # Prior standard deviation on log odds scale
  r <- mean(ys)
  n <- ( (1 / prior_sd^2) * ( (4 / r) + (4 / (1 - r)) ) )
  # In case of analyses with either no events or only events in all participants
  # n will be infinite regardless of the prior; in that case, n is set to 1,
  # just to ensure that simulations will not stop with an error if this occurs
  # due to chance in an early analysis
  if (!is.finite(n)) n <- 1
  # Prepare list to return posterior draws
  draws <- list()
  # Loop through all arms and generate posterior draws
  for (arm in arms) {
    ii <- which(allocs == arm) # Indices of current arm
    n_events <- sum(ys[ii]) # Total events in current arm
    draws[[arm]] <- rbeta(n_draws, # Posterior draws
                          (n * r / 2) + n_events,
                          (n * (1 - r) / 2) + length(ii) - n_events)
  }
  # Return draws after binding to matrix
  do.call(cbind, draws)
}
```

Trial design specification

Following this, the trial design is specified using the function defined above and a custom function for generating outcomes:

```
design_custom_prior_no_difference <- setup_trial(
  # Arms and scenario
  arms = c("Arm A", "Arm B"),
  true_ys = c(0.25, 0.25),
  control = NULL,
  highest_is_best = FALSE,
  # Custom outcome generation function largely matching the default one used in
  # setup_trial_binom()
  fun_y_gen = function(allocs) {
    # Setup empty return vector with outcomes
    y <- rep(NA, length(allocs))
    # Named numeric vector with event probabilities in each arm (for easy changing)
    event_probs <- c("Arm A" = 0.25, "Arm B" = 0.25)
    # Generate outcomes for each arm
    for (arm in c("Arm A", "Arm B")) {
      ii <- which(allocs == arm) # Indices of participants in current arm
      y[ii] <- rbinom(n = length(ii), size = 1, prob = event_probs[arm])
    }
    # Return outcomes
    y
  },
  # Custom function to calculate and return posterior draws (defined above)
  fun_draws = fun_draws_prior,
  # Allocation rules (response-adaptive randomisation with minimum limits)
  start_probs = c(0.5, 0.5),
  min_probs = c(1/3, 1/3),
  # Participants with data available/randomised at each analysis
  # Lag of 350 participants, maximum values in both arguments should match, and
  # the length of both vectors must match (pmin truncates values >10000)
  data_looks = seq(from = 1000, to = 10000, by = 500),
  randomised_at_looks = pmin(seq(from = 1000, to = 10000, by = 500) + 350, 10000),
  # Stopping rules
  inferiority = 0.01,
  superiority = 0.99,
  equivalence_prob = 0.9,
  equivalence_diff = 0.025,
  # Posterior draws
  n_draws = 10000,
  robust = TRUE
)
```

Log date and session info

Save date and R/package versions for reproducibility:

```
date()

## [1] "Mon Aug 25 09:43:04 2025"
```

```
sessionInfo()

## R version 4.4.1 (2024-06-14 ucrt)
## Platform: x86_64-w64-mingw32/x64
## Running under: Windows 11 x64 (build 22631)
##
## Matrix products: default
##
##
## locale:
## [1] LC_COLLATE=Danish_Denmark.utf8  LC_CTYPE=Danish_Denmark.utf8
## [3] LC_MONETARY=Danish_Denmark.utf8 LC_NUMERIC=C
## [5] LC_TIME=Danish_Denmark.utf8
##
## time zone: Europe/Copenhagen
## tzcode source: internal
##
## attached base packages:
## [1] stats     graphics  grDevices utils     datasets  methods   base
##
## other attached packages:
## [1] ggplot2_3.5.2 adaptr_1.4.0
##
## loaded via a namespace (and not attached):
##  [1] vctrs_0.6.5        cli_3.6.5          knitr_1.50         rlang_1.1.6
##  [5] xfun_0.52          generics_0.1.4     labeling_0.4.3     glue_1.8.0
##  [9] htmltools_0.5.8.1  scales_1.4.0       rmarkdown_2.29     grid_4.4.1
## [13] evaluate_1.0.4     tibble_3.3.0       fastmap_1.2.0      yaml_2.3.10
## [17] lifecycle_1.0.4    compiler_4.4.1     dplyr_1.1.4        RColorBrewer_1.1-3
## [21] pkgconfig_2.0.3    rstudioapi_0.17.1  farver_2.1.2       digest_0.6.37
## [25] R6_2.6.1           tidyselect_1.2.1   pillar_1.11.0      parallel_4.4.1
## [29] magrittr_2.0.3     withr_3.0.2        tools_4.4.1        gtable_0.3.6
```
